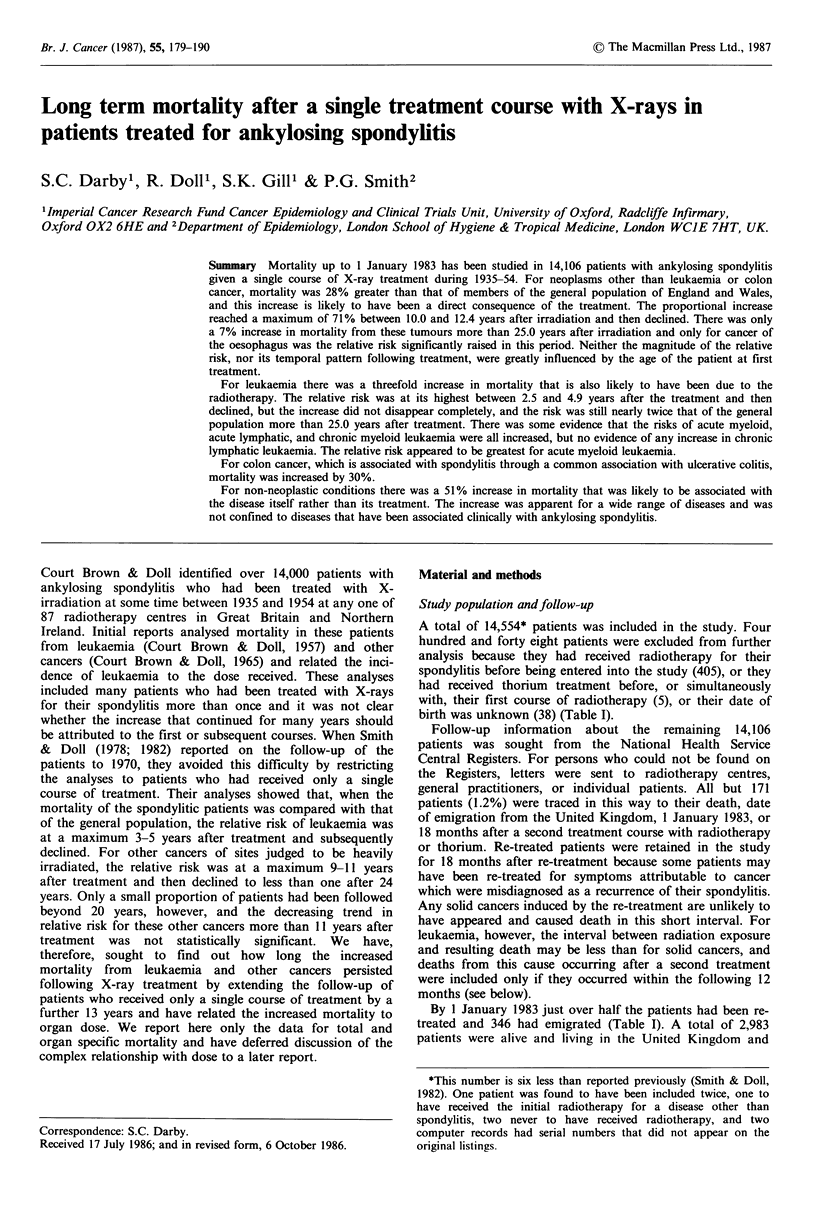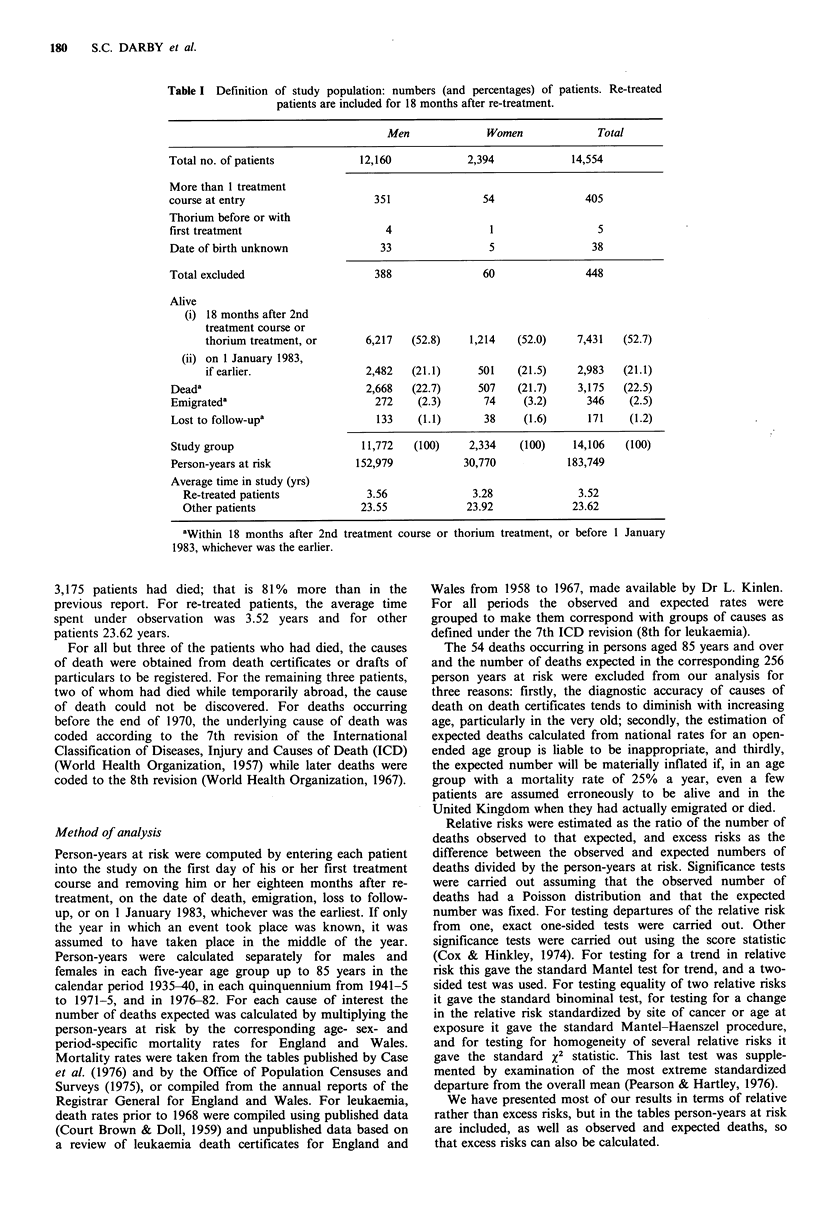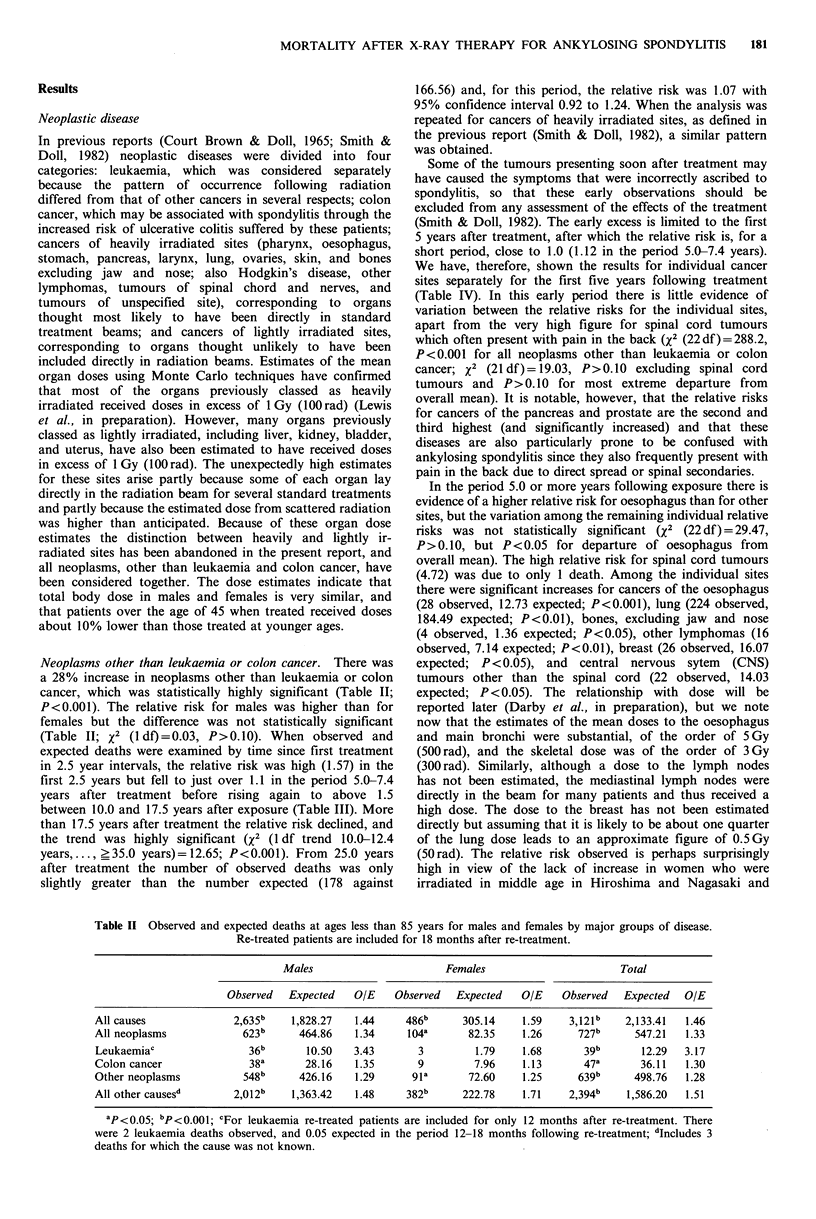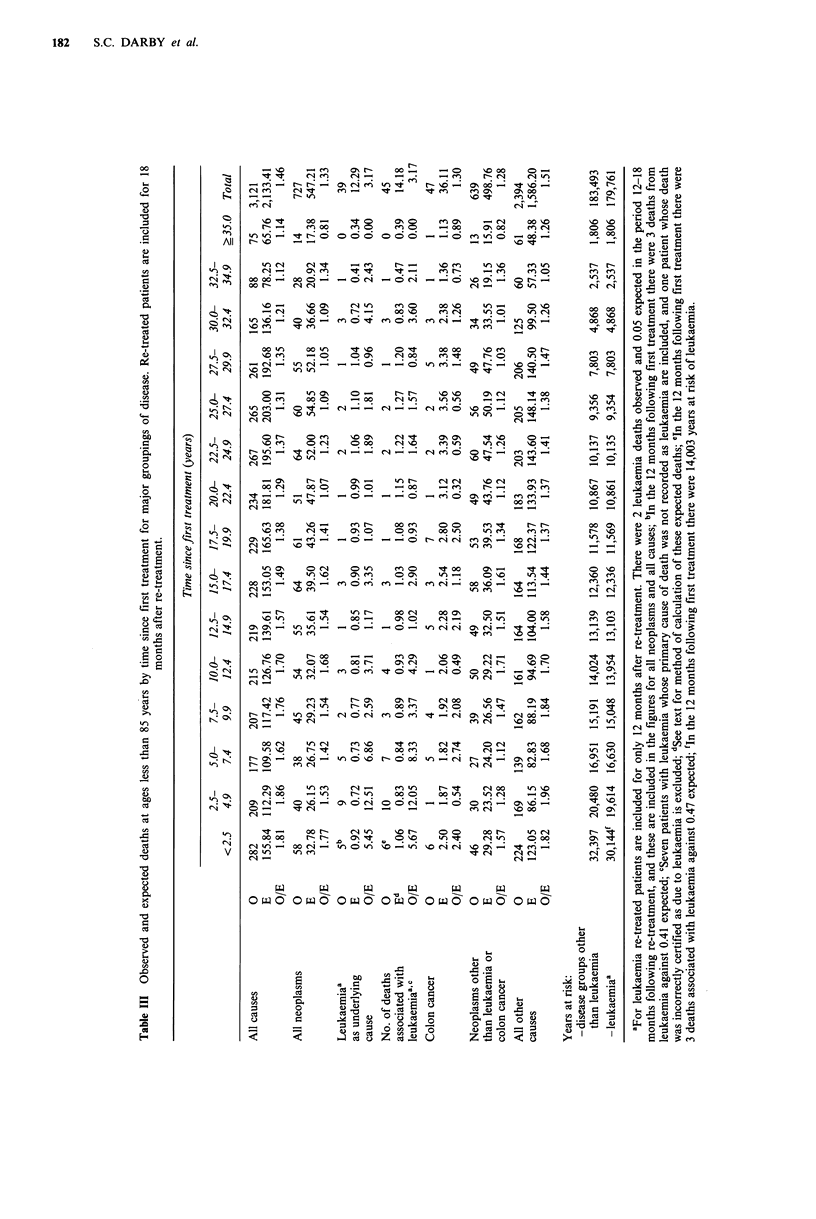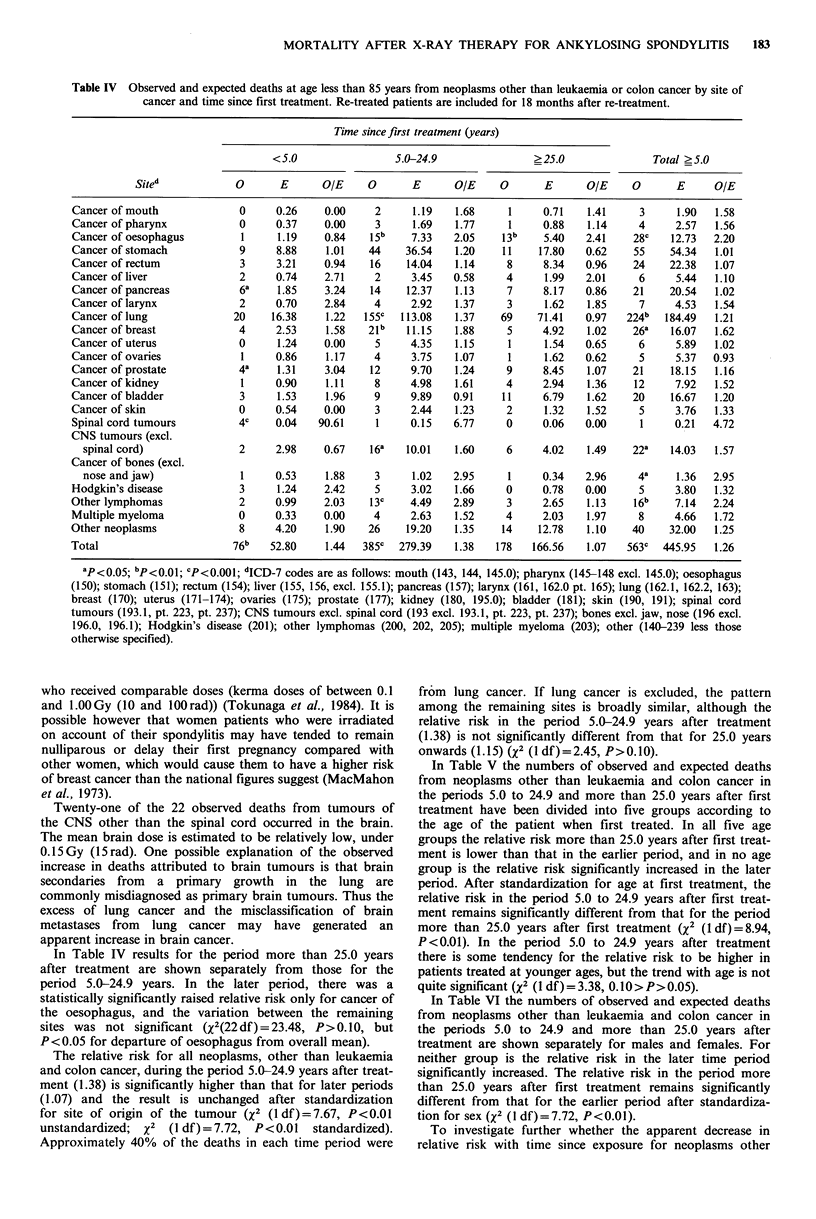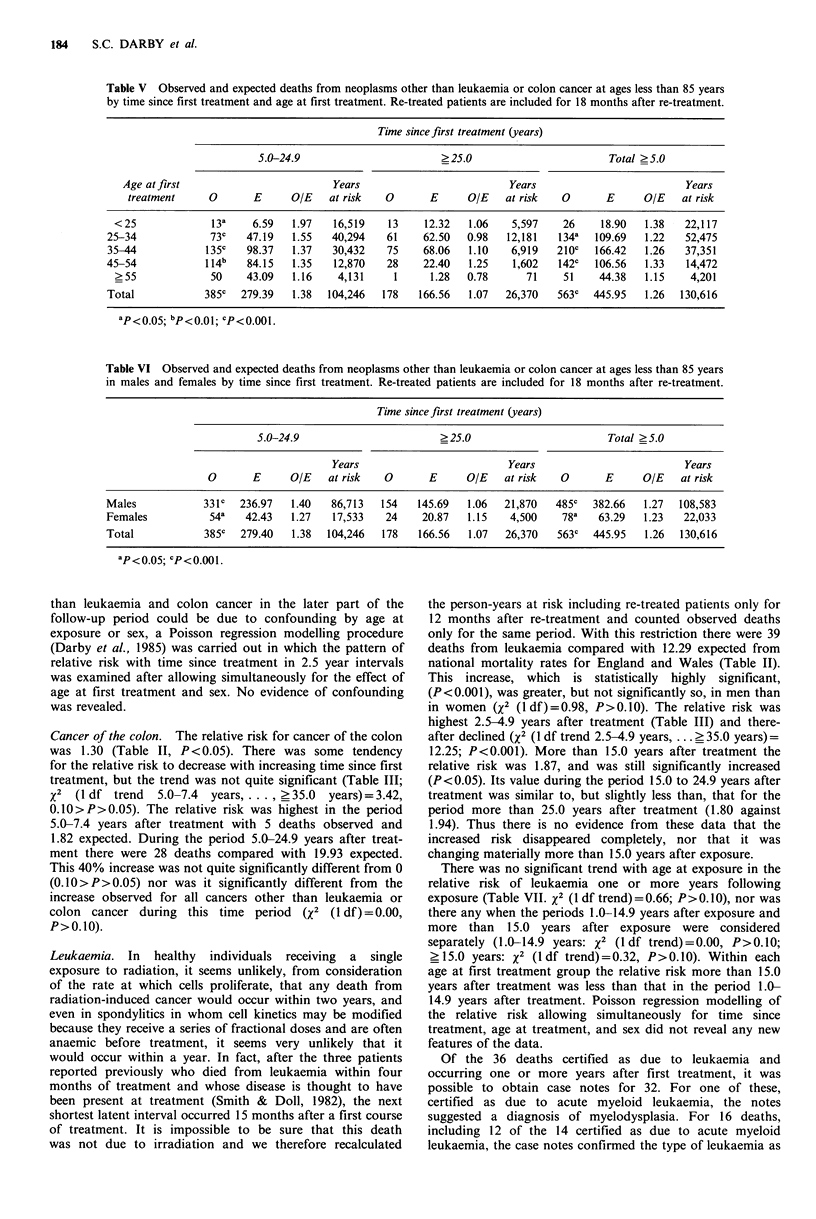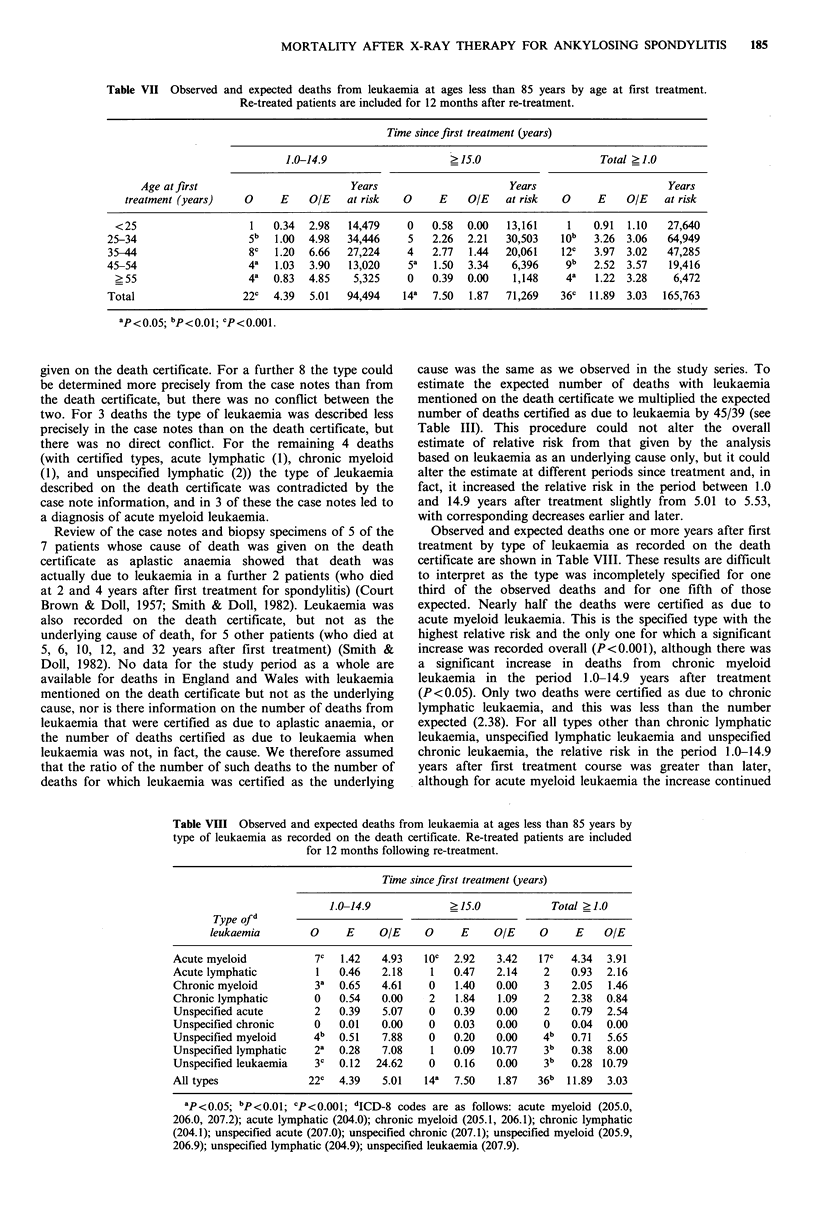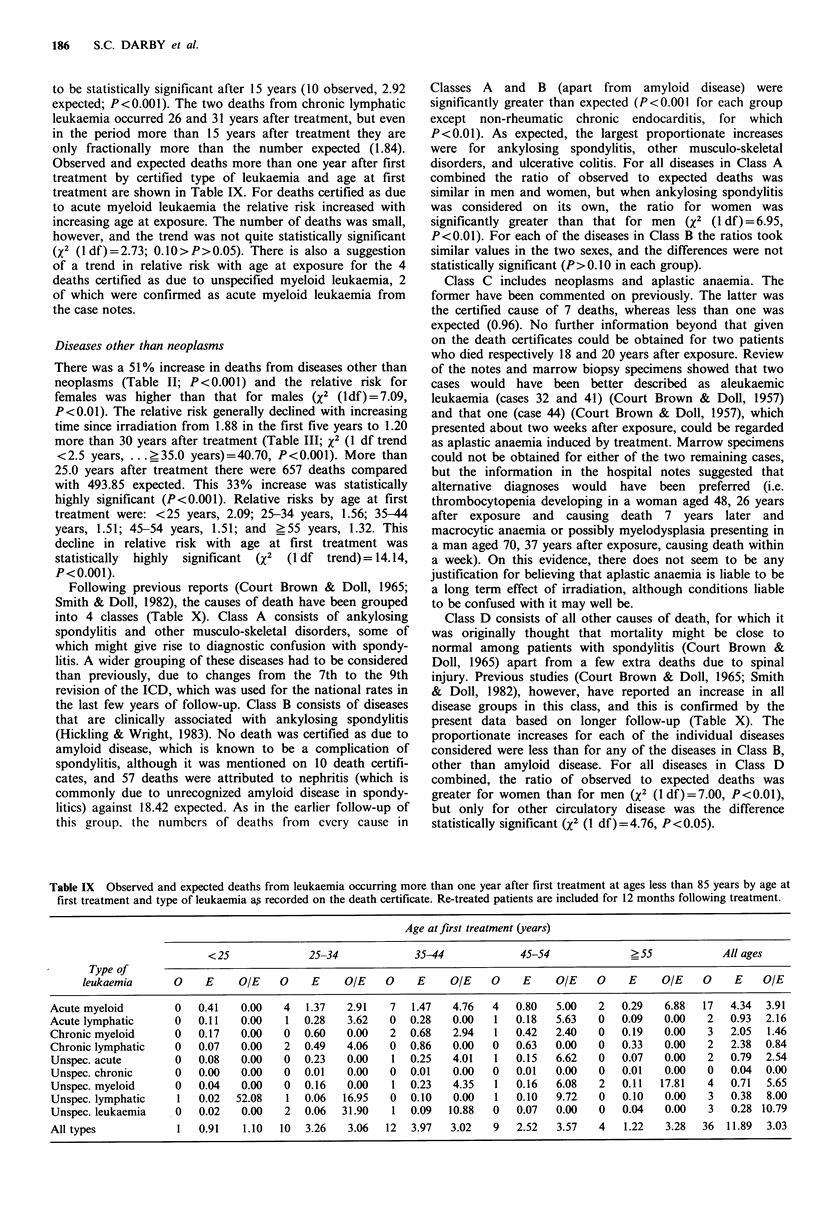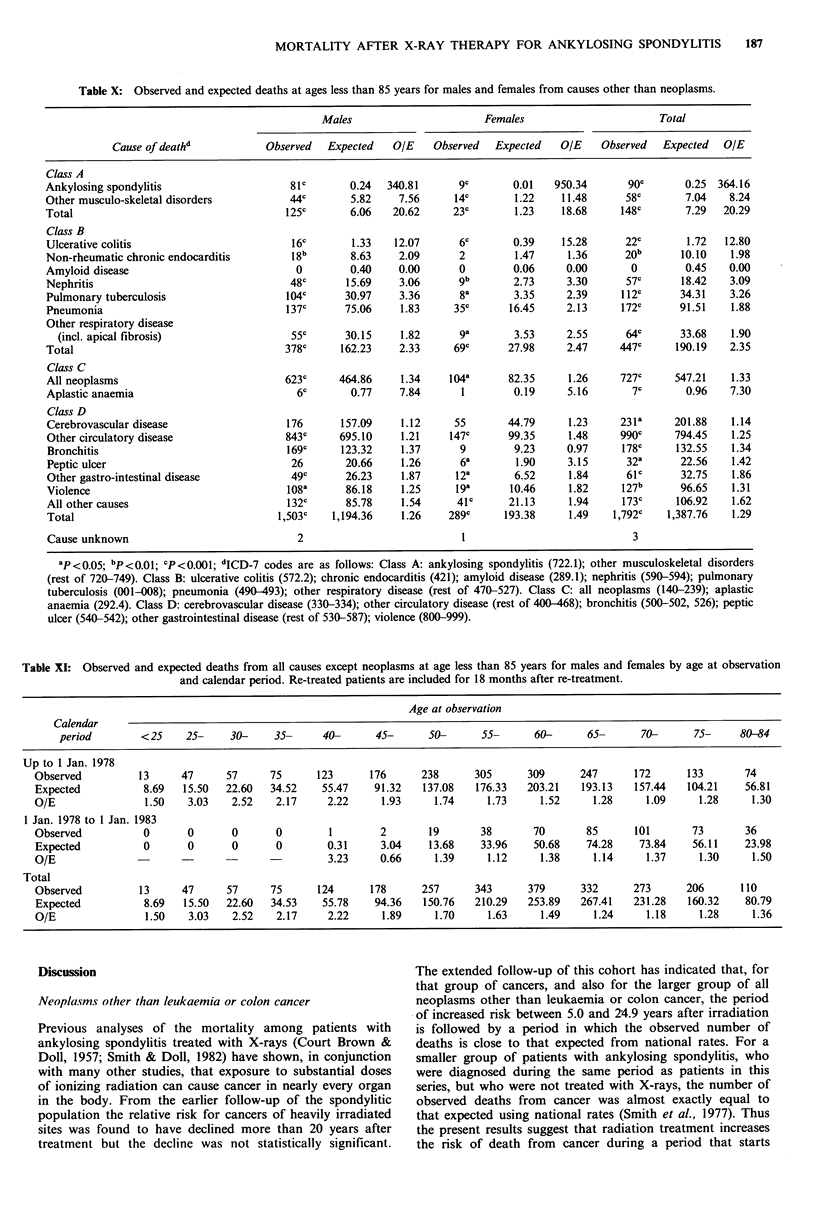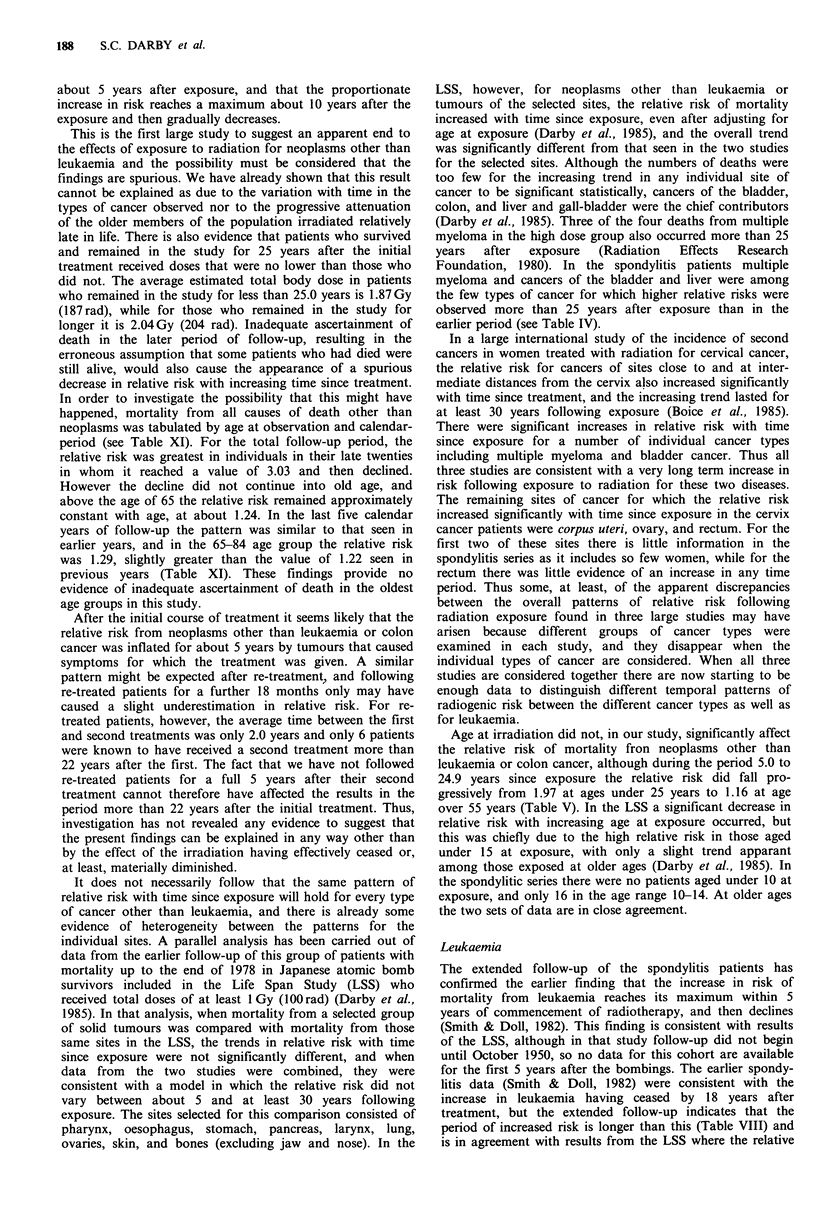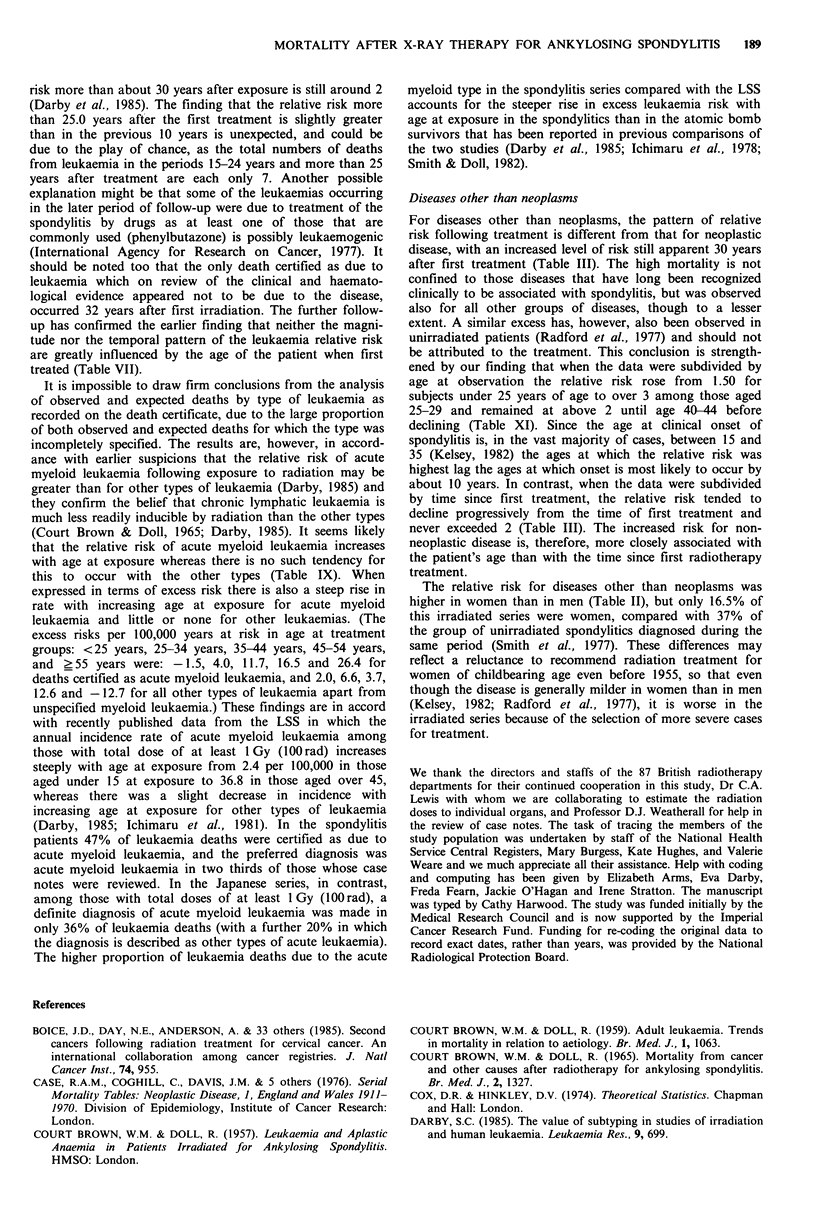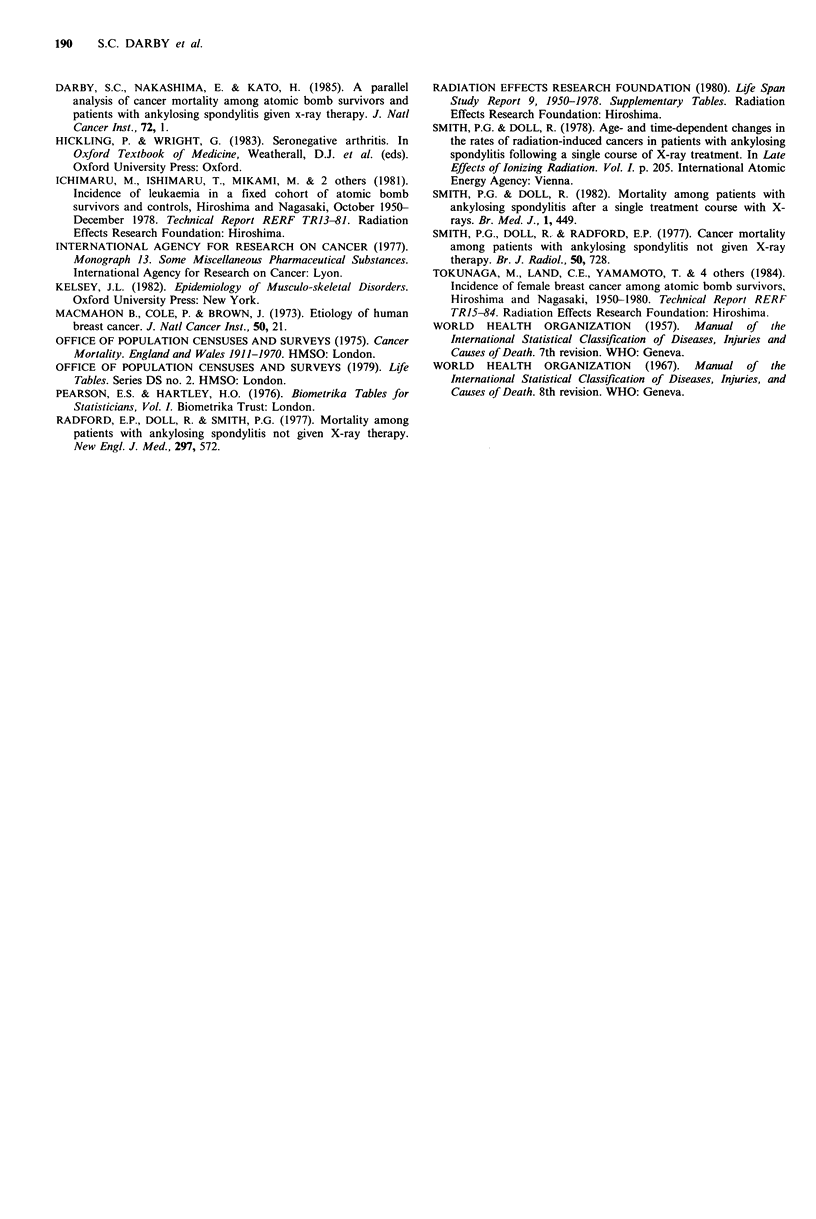# Long term mortality after a single treatment course with X-rays in patients treated for ankylosing spondylitis.

**DOI:** 10.1038/bjc.1987.35

**Published:** 1987-02

**Authors:** S. C. Darby, R. Doll, S. K. Gill, P. G. Smith

## Abstract

Mortality up to 1 January 1983 has been studied in 14,106 patients with ankylosing spondylitis given a single course of X-ray treatment during 1935-54. For neoplasms other than leukaemia or colon cancer, mortality was 28% greater than that of members of the general population of England and Wales, and this increase is likely to have been a direct consequence of the treatment. The proportional increase reached a maximum of 71% between 10.0 and 12.4 years after irradiation and then declined. There was only a 7% increase in mortality from these tumours more than 25.0 years after irradiation and only for cancer of the oesophagus was the relative risk significantly raised in this period. Neither the magnitude of the relative risk, nor its temporal pattern following treatment, were greatly influenced by the age of the patient at first treatment. For leukaemia there was a threefold increase in mortality that is also likely to have been due to the radiotherapy. The relative risk was at its highest between 2.5 and 4.9 years after the treatment and then declined, but the increase did not disappear completely, and the risk was still nearly twice that of the general population more than 25.0 years after treatment. There was some evidence that the risks of acute myeloid, acute lymphatic, and chronic myeloid leukaemia were all increased, but no evidence of any increase in chronic lymphatic leukaemia. The relative risk appeared to be greatest for acute myeloid leukaemia. For colon cancer, which is associated with spondylitis through a common association with ulcerative colitis, mortality was increased by 30%. For non-neoplastic conditions there was a 51% increase in mortality that was likely to be associated with the disease itself rather than its treatment. The increase was apparent for a wide range of diseases and was not confined to diseases that have been associated clinically with ankylosing spondylitis.


*Br. J. Cancer* (1987), **55**, 179–190 © The Macmillan Press Ltd., 1987

### Long term mortality after a single treatment course with X-rays in patients treated for ankylosing spondylitis

S.C. Darby[1], R. Doll[1], S.K. Gill[1] & P.G. Smith[2]

[1]*Imperial Cancer Research Fund Cancer Epidemiology and Clinical Trials Unit, University of Oxford, Radcliffe Infirmary, Oxford OX2 6HE and* [2]*Department of Epidemiology, London School of Hygiene & Tropical Medicine, London WC1E 7HT, UK.*

**Summary** Mortality up to 1 January 1983 has been studied in 14,106 patients with ankylosing spondylitis given a single course of X-ray treatment during 1935–54. For neoplasms other than leukaemia or colon cancer, mortality was 28% greater than that of members of the general population of England and Wales, and this increase is likely to have been a direct consequence of the treatment. The proportional increase reached a maximum of 71% between 10.0 and 12.4 years after irradiation and then declined. There was only a 7% increase in mortality from these tumours more than 25.0 years after irradiation and only for cancer of the oesophagus was the relative risk significantly raised in this period. Neither the magnitude of the relative risk, nor its temporal pattern following treatment, were greatly influenced by the age of the patient at first treatment.

For leukaemia there was a threefold increase in mortality that is also likely to have been due to the radiotherapy. The relative risk was at its highest between 2.5 and 4.9 years after the treatment and then declined, but the increase did not disappear completely, and the risk was still nearly twice that of the general population more than 25.0 years after treatment. There was some evidence that the risks of acute myeloid, acute lymphatic, and chronic myeloid leukaemia were all increased, but no evidence of any increase in chronic lymphatic leukaemia. The relative risk appeared to be greatest for acute myeloid leukaemia.

For colon cancer, which is associated with spondylitis through a common association with ulcerative colitis, mortality was increased by 30%.

For non-neoplastic conditions there was a 51% increase in mortality that was likely to be associated with the disease itself rather than its treatment. The increase was apparent for a wide range of diseases and was not confined to diseases that have been associated clinically with ankylosing spondylitis.

Court Brown & Doll identified over 14,000 patients with ankylosing spondylitis who had been treated with X-irradiation at some time between 1935 and 1954 at any one of 87 radiotherapy centres in Great Britain and Northern Ireland. Initial reports analysed mortality in these patients from leukaemia (Court Brown & Doll, 1957) and other cancers (Court Brown & Doll, 1965) and related the incidence of leukaemia to the dose received. These analyses included many patients who had been treated with X-rays for their spondylitis more than once and it was not clear whether the increase that continued for many years should be attributed to the first or subsequent courses. When Smith & Doll (1978; 1982) reported on the follow-up of the patients to 1970, they avoided this difficulty by restricting the analyses to patients who had received only a single course of treatment. Their analyses showed that, when the mortality of the spondylitic patients was compared with that of the general population, the relative risk of leukaemia was at a maximum 3–5 years after treatment and subsequently declined. For other cancers of sites judged to be heavily irradiated, the relative risk was at a maximum 9–11 years after treatment and then declined to less than one after 24 years. Only a small proportion of patients had been followed beyond 20 years, however, and the decreasing trend in relative risk for these other cancers more than 11 years after treatment was not statistically significant. We have, therefore, sought to find out how long the increased mortality from leukaemia and other cancers persisted following X-ray treatment by extending the follow-up of patients who received only a single course of treatment by a further 13 years and have related the increased mortality to organ dose. We report here only the data for total and organ specific mortality and have deferred discussion of the complex relationship with dose to a later report.

Correspondence: S.C. Darby.
Received 17 July 1986; and in revised form, 6 October 1986.

### Material and methods

### *Study population and follow-up*

A total of 14,554* patients was included in the study. Four hundred and forty eight patients were excluded from further analysis because they had received radiotherapy for their spondylitis before being entered into the study (405), or they had received thorium treatment before, or simultaneously with, their first course of radiotherapy (5), or their date of birth was unknown (38) (Table I).

Follow-up information about the remaining 14,106 patients was sought from the National Health Service Central Registers. For persons who could not be found on the Registers, letters were sent to radiotherapy centres, general practitioners, or individual patients. All but 171 patients (1.2%) were traced in this way to their death, date of emigration from the United Kingdom, 1 January 1983, or 18 months after a second treatment course with radiotherapy or thorium. Re-treated patients were retained in the study for 18 months after re-treatment because some patients may have been re-treated for symptoms attributable to cancer which were misdiagnosed as a recurrence of their spondylitis. Any solid cancers induced by the re-treatment are unlikely to have appeared and caused death in this short interval. For leukaemia, however, the interval between radiation exposure and resulting death may be less than for solid cancers, and deaths from this cause occurring after a second treatment were included only if they occurred within the following 12 months (see below).

By 1 January 1983 just over half the patients had been re-treated and 346 had emigrated (Table I). A total of 2,983 patients were alive and living in the United Kingdom and

*This number is six less than reported previously (Smith & Doll, 1982). One patient was found to have been included twice, one to have received the initial radiotherapy for a disease other than spondylitis, two never to have received radiotherapy, and two computer records had serial numbers that did not appear on the original listings.

**Table I** Definition of study population: numbers (and percentages) of patients. Re-treated patients are included for 18 months after re-treatment.

| | *Men* | | *Women* | | *Total* | |
|---|---|---|---|---|---|---|
| Total no. of patients | 12,160 | | 2,394 | | 14,554 | |
| More than 1 treatment course at entry | 351 | | 54 | | 405 | |
| Thorium before or with first treatment | 4 | | 1 | | 5 | |
| Date of birth unknown | 33 | | 5 | | 38 | |
| Total excluded | 388 | | 60 | | 448 | |
| Alive | | | | | | |
| (i) 18 months after 2nd treatment course or thorium treatment, or | 6,217 | (52.8) | 1,214 | (52.0) | 7,431 | (52.7) |
| (ii) on 1 January 1983, if earlier. | 2,482 | (21.1) | 501 | (21.5) | 2,983 | (21.1) |
| Dead[a] | 2,668 | (22.7) | 507 | (21.7) | 3,175 | (22.5) |
| Emigrated[a] | 272 | (2.3) | 74 | (3.2) | 346 | (2.5) |
| Lost to follow-up[a] | 133 | (1.1) | 38 | (1.6) | 171 | (1.2) |
| Study group | 11,772 | (100) | 2,334 | (100) | 14,106 | (100) |
| Person-years at risk | 152,979 | | 30,770 | | 183,749 | |
| Average time in study (yrs) | | | | | | |
| Re-treated patients | 3.56 | | 3.28 | | 3.52 | |
| Other patients | 23.55 | | 23.92 | | 23.62 | |

[a]Within 18 months after 2nd treatment course or thorium treatment, or before 1 January 1983, whichever was the earlier.

3,175 patients had died; that is 81% more than in the previous report. For re-treated patients, the average time spent under observation was 3.52 years and for other patients 23.62 years.

For all but three of the patients who had died, the causes of death were obtained from death certificates or drafts of particulars to be registered. For the remaining three patients, two of whom had died while temporarily abroad, the cause of death could not be discovered. For deaths occurring before the end of 1970, the underlying cause of death was coded according to the 7th revision of the International Classification of Diseases, Injury and Causes of Death (ICD) (World Health Organization, 1957) while later deaths were coded to the 8th revision (World Health Organization, 1967).

### *Method of analysis*

Person-years at risk were computed by entering each patient into the study on the first day of his or her first treatment course and removing him or her eighteen months after re-treatment, on the date of death, emigration, loss to follow-up, or on 1 January 1983, whichever was the earliest. If only the year in which an event took place was known, it was assumed to have taken place in the middle of the year. Person-years were calculated separately for males and females in each five-year age group up to 85 years in the calendar period 1935–40, in each quinquennium from 1941–5 to 1971–5, and in 1976–82. For each cause of interest the number of deaths expected was calculated by multiplying the person-years at risk by the corresponding age- sex- and period-specific mortality rates for England and Wales. Mortality rates were taken from the tables published by Case *et al.* (1976) and by the Office of Population Censuses and Surveys (1975), or compiled from the annual reports of the Registrar General for England and Wales. For leukaemia, death rates prior to 1968 were compiled using published data (Court Brown & Doll, 1959) and unpublished data based on a review of leukaemia death certificates for England and Wales from 1958 to 1967, made available by Dr L. Kinlen. For all periods the observed and expected rates were grouped to make them correspond with groups of causes as defined under the 7th ICD revision (8th for leukaemia).

The 54 deaths occurring in persons aged 85 years and over and the number of deaths expected in the corresponding 256 person years at risk were excluded from our analysis for three reasons: firstly, the diagnostic accuracy of causes of death on death certificates tends to diminish with increasing age, particularly in the very old; secondly, the estimation of expected deaths calculated from national rates for an open-ended age group is liable to be inappropriate, and thirdly, the expected number will be materially inflated if, in an age group with a mortality rate of 25% a year, even a few patients are assumed erroneously to be alive and in the United Kingdom when they had actually emigrated or died.

Relative risks were estimated as the ratio of the number of deaths observed to that expected, and excess risks as the difference between the observed and expected numbers of deaths divided by the person-years at risk. Significance tests were carried out assuming that the observed number of deaths had a Poisson distribution and that the expected number was fixed. For testing departures of the relative risk from one, exact one-sided tests were carried out. Other significance tests were carried out using the score statistic (Cox & Hinkley, 1974). For testing for a trend in relative risk this gave the standard Mantel test for trend, and a two-sided test was used. For testing equality of two relative risks it gave the standard binominal test, for testing for a change in the relative risk standardized by site of cancer or age at exposure it gave the standard Mantel–Haenszel procedure, and for testing for homogeneity of several relative risks it gave the standard $\chi^2$ statistic. This last test was supplemented by examination of the most extreme standardized departure from the overall mean (Pearson & Hartley, 1976).

We have presented most of our results in terms of relative rather than excess risks, but in the tables person-years at risk are included, as well as observed and expected deaths, so that excess risks can also be calculated.

### Results

### *Neoplastic disease*

In previous reports (Court Brown & Doll, 1965; Smith & Doll, 1982) neoplastic diseases were divided into four categories: leukaemia, which was considered separately because the pattern of occurrence following radiation differed from that of other cancers in several respects; colon cancer, which may be associated with spondylitis through the increased risk of ulcerative colitis suffered by these patients; cancers of heavily irradiated sites (pharynx, oesophagus, stomach, pancreas, larynx, lung, ovaries, skin, and bones excluding jaw and nose; also Hodgkin's disease, other lymphomas, tumours of spinal chord and nerves, and tumours of unspecified site), corresponding to organs thought most likely to have been directly in standard treatment beams; and cancers of lightly irradiated sites, corresponding to organs thought unlikely to have been included directly in radiation beams. Estimates of the mean organ doses using Monte Carlo techniques have confirmed that most of the organs previously classed as heavily irradiated received doses in excess of 1 Gy (100 rad) (Lewis *et al.*, in preparation). However, many organs previously classed as lightly irradiated, including liver, kidney, bladder, and uterus, have also been estimated to have received doses in excess of 1 Gy (100 rad). The unexpectedly high estimates for these sites arise partly because some of each organ lay directly in the radiation beam for several standard treatments and partly because the estimated dose from scattered radiation was higher than anticipated. Because of these organ dose estimates the distinction between heavily and lightly irradiated sites has been abandoned in the present report, and all neoplasms, other than leukaemia and colon cancer, have been considered together. The dose estimates indicate that total body dose in males and females is very similar, and that patients over the age of 45 when treated received doses about 10% lower than those treated at younger ages.

*Neoplasms other than leukaemia or colon cancer.* There was a 28% increase in neoplasms other than leukaemia or colon cancer, which was statistically highly significant (Table II; $P<0.001$). The relative risk for males was higher than for females but the difference was not statistically significant (Table II; $\chi^2$ (1 df) = 0.03, $P>0.10$). When observed and expected deaths were examined by time since first treatment in 2.5 year intervals, the relative risk was high (1.57) in the first 2.5 years but fell to just over 1.1 in the period 5.0–7.4 years after treatment before rising again to above 1.5 between 10.0 and 17.5 years after exposure (Table III). More than 17.5 years after treatment the relative risk declined, and the trend was highly significant ($\chi^2$ (1 df trend 10.0–12.4 years,..., ≧35.0 years) = 12.65; $P<0.001$). From 25.0 years after treatment the number of observed deaths was only slightly greater than the number expected (178 against 166.56) and, for this period, the relative risk was 1.07 with 95% confidence interval 0.92 to 1.24. When the analysis was repeated for cancers of heavily irradiated sites, as defined in the previous report (Smith & Doll, 1982), a similar pattern was obtained.

Some of the tumours presenting soon after treatment may have caused the symptoms that were incorrectly ascribed to spondylitis, so that these early observations should be excluded from any assessment of the effects of the treatment (Smith & Doll, 1982). The early excess is limited to the first 5 years after treatment, after which the relative risk is, for a short period, close to 1.0 (1.12 in the period 5.0–7.4 years). We have, therefore, shown the results for individual cancer sites separately for the first five years following treatment (Table IV). In this early period there is little evidence of variation between the relative risks for the individual sites, apart from the very high figure for spinal cord tumours which often present with pain in the back ($\chi^2$ (22 df) = 288.2, $P<0.001$ for all neoplasms other than leukaemia or colon cancer; $\chi^2$ (21 df) = 19.03, $P>0.10$ excluding spinal cord tumours and $P>0.10$ for most extreme departure from overall mean). It is notable, however, that the relative risks for cancers of the pancreas and prostate are the second and third highest (and significantly increased) and that these diseases are also particularly prone to be confused with ankylosing spondylitis since they also frequently present with pain in the back due to direct spread or spinal secondaries.

In the period 5.0 or more years following exposure there is evidence of a higher relative risk for oesophagus than for other sites, but the variation among the remaining individual relative risks was not statistically significant ($\chi^2$ (22 df) = 29.47, $P>0.10$, but $P<0.05$ for departure of oesophagus from overall mean). The high relative risk for spinal cord tumours (4.72) was due to only 1 death. Among the individual sites there were significant increases for cancers of the oesophagus (28 observed, 12.73 expected; $P<0.001$), lung (224 observed, 184.49 expected; $P<0.01$), bones, excluding jaw and nose (4 observed, 1.36 expected; $P<0.05$), other lymphomas (16 observed, 7.14 expected; $P<0.01$), breast (26 observed, 16.07 expected; $P<0.05$), and central nervous sytem (CNS) tumours other than the spinal cord (22 observed, 14.03 expected; $P<0.05$). The relationship with dose will be reported later (Darby *et al.*, in preparation), but we note now that the estimates of the mean doses to the oesophagus and main bronchi were substantial, of the order of 5 Gy (500 rad), and the skeletal dose was of the order of 3 Gy (300 rad). Similarly, although a dose to the lymph nodes has not been estimated, the mediastinal lymph nodes were directly in the beam for many patients and thus received a high dose. The dose to the breast has not been estimated directly but assuming that it is likely to be about one quarter of the lung dose leads to an approximate figure of 0.5 Gy (50 rad). The relative risk observed is perhaps surprisingly high in view of the lack of increase in women who were irradiated in middle age in Hiroshima and Nagasaki and

**Table II** Observed and expected deaths at ages less than 85 years for males and females by major groups of disease. Re-treated patients are included for 18 months after re-treatment.

| | *Males* | | | *Females* | | | *Total* | | |
|---|---|---|---|---|---|---|---|---|---|
| | *Observed* | *Expected* | *O/E* | *Observed* | *Expected* | *O/E* | *Observed* | *Expected* | *O/E* |
| All causes | 2,635[b] | 1,828.27 | 1.44 | 486[b] | 305.14 | 1.59 | 3,121[b] | 2,133.41 | 1.46 |
| All neoplasms | 623[b] | 464.86 | 1.34 | 104[a] | 82.35 | 1.26 | 727[b] | 547.21 | 1.33 |
| Leukaemia[c] | 36[b] | 10.50 | 3.43 | 3 | 1.79 | 1.68 | 39[b] | 12.29 | 3.17 |
| Colon cancer | 38[a] | 28.16 | 1.35 | 9 | 7.96 | 1.13 | 47[a] | 36.11 | 1.30 |
| Other neoplasms | 548[b] | 426.16 | 1.29 | 91[a] | 72.60 | 1.25 | 639[b] | 498.76 | 1.28 |
| All other causes[d] | 2,012[b] | 1,363.42 | 1.48 | 382[b] | 222.78 | 1.71 | 2,394[b] | 1,586.20 | 1.51 |

[a]$P<0.05$; [b]$P<0.001$; [c]For leukaemia re-treated patients are included for only 12 months after re-treatment. There were 2 leukaemia deaths observed, and 0.05 expected in the period 12–18 months following re-treatment; [d]Includes 3 deaths for which the cause was not known.

**Table III** Observed and expected deaths at ages less than 85 years by time since first treatment for major groupings of disease. Re-treated patients are included for 18 months after re-treatment.

| | | Time since first treatment (years) | | | | | | | | | | | | | | | |
|---|---|---|---|---|---|---|---|---|---|---|---|---|---|---|---|---|---|
| | | <2.5 | 2.5–4.9 | 5.0–7.4 | 7.5–9.9 | 10.0–12.4 | 12.5–14.9 | 15.0–17.4 | 17.5–19.9 | 20.0–22.4 | 22.5–24.9 | 25.0–27.4 | 27.5–29.9 | 30.0–32.4 | 32.5–34.9 | ≧35.0 | Total |
| All causes | O | 282 | 209 | 177 | 207 | 215 | 219 | 228 | 229 | 234 | 267 | 265 | 261 | 165 | 88 | 75 | 3,121 |
| | E | 155.84 | 112.29 | 109.58 | 117.42 | 126.76 | 139.61 | 153.05 | 165.63 | 181.81 | 195.60 | 203.00 | 192.68 | 136.16 | 78.25 | 65.76 | 2,133.41 |
| | O/E | 1.81 | 1.86 | 1.62 | 1.76 | 1.70 | 1.57 | 1.49 | 1.38 | 1.29 | 1.37 | 1.31 | 1.35 | 1.21 | 1.12 | 1.14 | 1.46 |
| All neoplasms | O | 58 | 40 | 38 | 45 | 54 | 55 | 64 | 61 | 51 | 64 | 60 | 55 | 40 | 28 | 14 | 727 |
| | E | 32.78 | 26.15 | 26.75 | 29.23 | 32.07 | 35.61 | 39.50 | 43.26 | 47.87 | 52.00 | 54.85 | 52.18 | 36.66 | 20.92 | 17.38 | 547.21 |
| | O/E | 1.77 | 1.53 | 1.42 | 1.54 | 1.68 | 1.54 | 1.62 | 1.41 | 1.07 | 1.23 | 1.09 | 1.05 | 1.09 | 1.34 | 0.81 | 1.33 |
| Leukaemia[a] as underlying cause | O | 5[b] | 9 | 5 | 2 | 3 | 1 | 3 | 1 | 1 | 2 | 2 | 1 | 3 | 1 | 0 | 39 |
| | E | 0.92 | 0.72 | 0.73 | 0.77 | 0.81 | 0.85 | 0.90 | 0.93 | 0.99 | 1.06 | 1.10 | 1.04 | 0.72 | 0.41 | 0.34 | 12.29 |
| | O/E | 5.45 | 12.51 | 6.86 | 2.59 | 3.71 | 1.17 | 3.35 | 1.07 | 1.01 | 1.89 | 1.81 | 0.96 | 4.15 | 2.43 | 0.00 | 3.17 |
| No. of deaths associated with leukaemia[a,c] | O | 6[e] | 10 | 7 | 3 | 4 | 1 | 3 | 1 | 1 | 2 | 2 | 1 | 3 | 1 | 0 | 45 |
| | E[d] | 1.06 | 0.83 | 0.84 | 0.89 | 0.93 | 0.98 | 1.03 | 1.08 | 1.15 | 1.22 | 1.27 | 1.20 | 0.83 | 0.47 | 0.39 | 14.18 |
| | O/E | 5.67 | 12.05 | 8.33 | 3.37 | 4.29 | 1.02 | 2.90 | 0.93 | 0.87 | 1.64 | 1.57 | 0.84 | 3.60 | 2.11 | 0.00 | 3.17 |
| Colon cancer | O | 6 | 1 | 5 | 4 | 1 | 5 | 3 | 7 | 1 | 2 | 2 | 5 | 3 | 1 | 1 | 47 |
| | E | 2.50 | 1.87 | 1.82 | 1.92 | 2.06 | 2.28 | 2.54 | 2.80 | 3.12 | 3.39 | 3.56 | 3.38 | 2.38 | 1.36 | 1.13 | 36.11 |
| | O/E | 2.40 | 0.54 | 2.74 | 2.08 | 0.49 | 2.19 | 1.18 | 2.50 | 0.32 | 0.59 | 0.56 | 1.48 | 1.26 | 0.73 | 0.89 | 1.30 |
| Neoplasms other than leukaemia or colon cancer | O | 46 | 30 | 27 | 39 | 50 | 49 | 58 | 53 | 49 | 60 | 56 | 49 | 34 | 26 | 13 | 639 |
| | E | 29.28 | 23.52 | 24.20 | 26.56 | 29.22 | 32.50 | 36.09 | 39.53 | 43.76 | 47.54 | 50.19 | 47.76 | 33.55 | 19.15 | 15.91 | 498.76 |
| | O/E | 1.57 | 1.28 | 1.12 | 1.47 | 1.71 | 1.51 | 1.61 | 1.34 | 1.12 | 1.26 | 1.12 | 1.03 | 1.01 | 1.36 | 0.82 | 1.28 |
| All other causes | O | 224 | 169 | 139 | 162 | 161 | 164 | 164 | 168 | 183 | 203 | 205 | 206 | 125 | 60 | 61 | 2,394 |
| | E | 123.05 | 86.15 | 82.83 | 88.19 | 94.69 | 104.00 | 113.54 | 122.37 | 133.93 | 143.60 | 148.14 | 140.50 | 99.50 | 57.33 | 48.38 | 1,586.20 |
| | O/E | 1.82 | 1.96 | 1.68 | 1.84 | 1.70 | 1.58 | 1.44 | 1.37 | 1.37 | 1.41 | 1.38 | 1.47 | 1.26 | 1.05 | 1.26 | 1.51 |
| Years at risk: –disease groups other than leukaemia | | 32,397 | 20,480 | 16,951 | 15,191 | 14,024 | 13,139 | 12,360 | 11,578 | 10,867 | 10,137 | 9,356 | 7,803 | 4,868 | 2,537 | 1,806 | 183,493 |
| –leukaemia[a] | | 30,144[f] | 19,614 | 16,630 | 15,048 | 13,954 | 13,103 | 12,336 | 11,569 | 10,861 | 10,135 | 9,354 | 7,803 | 4,868 | 2,537 | 1,806 | 179,761 |

[a]For leukaemia re-treated patients are included for only 12 months after re-treatment. There were 2 leukaemia deaths observed and 0.05 expected in the period 12–18 months following re-treatment, and these are included in the figures for all neoplasms and all causes; [b]In the 12 months following first treatment there were 3 deaths from leukaemia against 0.41 expected; [c]Seven patients with leukaemia whose primary cause of death was not recorded as leukaemia are included, and one patient whose death was incorrectly certified as due to leukaemia is excluded; [d]See text for method of calculation of these expected deaths; [e]In the 12 months following first treatment there were 3 deaths associated with leukaemia against 0.47 expected; [f]In the 12 months following first treatment there were 14,003 years at risk of leukaemia.

**Table IV** Observed and expected deaths at age less than 85 years from neoplasms other than leukaemia or colon cancer by site of cancer and time since first treatment. Re-treated patients are included for 18 months after re-treatment.

| | *Time since first treatment (years)* | | | | | | | | | | | |
|---|---|---|---|---|---|---|---|---|---|---|---|---|
| | *<5.0* | | | *5.0–24.9* | | | *≧25.0* | | | *Total ≧5.0* | | |
| *Site*[d] | *O* | *E* | *O/E* | *O* | *E* | *O/E* | *O* | *E* | *O/E* | *O* | *E* | *O/E* |
| Cancer of mouth | 0 | 0.26 | 0.00 | 2 | 1.19 | 1.68 | 1 | 0.71 | 1.41 | 3 | 1.90 | 1.58 |
| Cancer of pharynx | 0 | 0.37 | 0.00 | 3 | 1.69 | 1.77 | 1 | 0.88 | 1.14 | 4 | 2.57 | 1.56 |
| Cancer of oesophagus | 1 | 1.19 | 0.84 | 15[b] | 7.33 | 2.05 | 13[b] | 5.40 | 2.41 | 28[c] | 12.73 | 2.20 |
| Cancer of stomach | 9 | 8.88 | 1.01 | 44 | 36.54 | 1.20 | 11 | 17.80 | 0.62 | 55 | 54.34 | 1.01 |
| Cancer of rectum | 3 | 3.21 | 0.94 | 16 | 14.04 | 1.14 | 8 | 8.34 | 0.96 | 24 | 22.38 | 1.07 |
| Cancer of liver | 2 | 0.74 | 2.71 | 2 | 3.45 | 0.58 | 4 | 1.99 | 2.01 | 6 | 5.44 | 1.10 |
| Cancer of pancreas | 6[a] | 1.85 | 3.24 | 14 | 12.37 | 1.13 | 7 | 8.17 | 0.86 | 21 | 20.54 | 1.02 |
| Cancer of larynx | 2 | 0.70 | 2.84 | 4 | 2.92 | 1.37 | 3 | 1.62 | 1.85 | 7 | 4.53 | 1.54 |
| Cancer of lung | 20 | 16.38 | 1.22 | 155[c] | 113.08 | 1.37 | 69 | 71.41 | 0.97 | 224[b] | 184.49 | 1.21 |
| Cancer of breast | 4 | 2.53 | 1.58 | 21[b] | 11.15 | 1.88 | 5 | 4.92 | 1.02 | 26[a] | 16.07 | 1.62 |
| Cancer of uterus | 0 | 1.24 | 0.00 | 5 | 4.35 | 1.15 | 1 | 1.54 | 0.65 | 6 | 5.89 | 1.02 |
| Cancer of ovaries | 1 | 0.86 | 1.17 | 4 | 3.75 | 1.07 | 1 | 1.62 | 0.62 | 5 | 5.37 | 0.93 |
| Cancer of prostate | 4[a] | 1.31 | 3.04 | 12 | 9.70 | 1.24 | 9 | 8.45 | 1.07 | 21 | 18.15 | 1.16 |
| Cancer of kidney | 1 | 0.90 | 1.11 | 8 | 4.98 | 1.61 | 4 | 2.94 | 1.36 | 12 | 7.92 | 1.52 |
| Cancer of bladder | 3 | 1.53 | 1.96 | 9 | 9.89 | 0.91 | 11 | 6.79 | 1.62 | 20 | 16.67 | 1.20 |
| Cancer of skin | 0 | 0.54 | 0.00 | 3 | 2.44 | 1.23 | 2 | 1.32 | 1.52 | 5 | 3.76 | 1.33 |
| Spinal cord tumours | 4[c] | 0.04 | 90.61 | 1 | 0.15 | 6.77 | 0 | 0.06 | 0.00 | 1 | 0.21 | 4.72 |
| CNS tumours (excl. spinal cord) | 2 | 2.98 | 0.67 | 16[a] | 10.01 | 1.60 | 6 | 4.02 | 1.49 | 22[a] | 14.03 | 1.57 |
| Cancer of bones (excl. nose and jaw) | 1 | 0.53 | 1.88 | 3 | 1.02 | 2.95 | 1 | 0.34 | 2.96 | 4[a] | 1.36 | 2.95 |
| Hodgkin's disease | 3 | 1.24 | 2.42 | 5 | 3.02 | 1.66 | 0 | 0.78 | 0.00 | 5 | 3.80 | 1.32 |
| Other lymphomas | 2 | 0.99 | 2.03 | 13[c] | 4.49 | 2.89 | 3 | 2.65 | 1.13 | 16[b] | 7.14 | 2.24 |
| Multiple myeloma | 0 | 0.33 | 0.00 | 4 | 2.63 | 1.52 | 4 | 2.03 | 1.97 | 8 | 4.66 | 1.72 |
| Other neoplasms | 8 | 4.20 | 1.90 | 26 | 19.20 | 1.35 | 14 | 12.78 | 1.10 | 40 | 32.00 | 1.25 |
| Total | 76[b] | 52.80 | 1.44 | 385[c] | 279.39 | 1.38 | 178 | 166.56 | 1.07 | 563[c] | 445.95 | 1.26 |

[a]$P<0.05$; [b]$P<0.01$; [c]$P<0.001$; [d]ICD-7 codes are as follows: mouth (143, 144, 145.0); pharynx (145–148 excl. 145.0); oesophagus (150); stomach (151); rectum (154); liver (155, 156, excl. 155.1); pancreas (157); larynx (161, 162.0 pt. 165); lung (162.1, 162.2, 163); breast (170); uterus (171–174); ovaries (175); prostate (177); kidney (180, 195.0); bladder (181); skin (190, 191); spinal cord tumours (193.1, pt. 223, pt. 237); CNS tumours excl. spinal cord (193 excl. 193.1, pt. 223, pt. 237); bones excl. jaw, nose (196 excl. 196.0, 196.1); Hodgkin's disease (201); other lymphomas (200, 202, 205); multiple myeloma (203); other (140–239 less those otherwise specified).

who received comparable doses (kerma doses of between 0.1 and 1.00 Gy (10 and 100 rad)) (Tokunaga *et al.*, 1984). It is possible however that women patients who were irradiated on account of their spondylitis may have tended to remain nulliparous or delay their first pregnancy compared with other women, which would cause them to have a higher risk of breast cancer than the national figures suggest (MacMahon *et al.*, 1973).

Twenty-one of the 22 observed deaths from tumours of the CNS other than the spinal cord occurred in the brain. The mean brain dose is estimated to be relatively low, under 0.15 Gy (15 rad). One possible explanation of the observed increase in deaths attributed to brain tumours is that brain secondaries from a primary growth in the lung are commonly misdiagnosed as primary brain tumours. Thus the excess of lung cancer and the misclassification of brain metastases from lung cancer may have generated an apparent increase in brain cancer.

In Table IV results for the period more than 25.0 years after treatment are shown separately from those for the period 5.0–24.9 years. In the later period, there was a statistically significantly raised relative risk only for cancer of the oesophagus, and the variation between the remaining sites was not significant ($\chi^2(22\,df)=23.48$, $P>0.10$, but $P<0.05$ for departure of oesophagus from overall mean).

The relative risk for all neoplasms, other than leukaemia and colon cancer, during the period 5.0–24.9 years after treatment (1.38) is significantly higher than that for later periods (1.07) and the result is unchanged after standardization for site of origin of the tumour ($\chi^2$ (1 df) = 7.67, $P<0.01$ unstandardized; $\chi^2$ (1 df) = 7.72, $P<0.01$ standardized). Approximately 40% of the deaths in each time period were from lung cancer. If lung cancer is excluded, the pattern among the remaining sites is broadly similar, although the relative risk in the period 5.0–24.9 years after treatment (1.38) is not significantly different from that for 25.0 years onwards (1.15) ($\chi^2$ (1 df) = 2.45, $P>0.10$).

In Table V the numbers of observed and expected deaths from neoplasms other than leukaemia and colon cancer in the periods 5.0 to 24.9 and more than 25.0 years after first treatment have been divided into five groups according to the age of the patient when first treated. In all five age groups the relative risk more than 25.0 years after first treatment is lower than that in the earlier period, and in no age group is the relative risk significantly increased in the later period. After standardization for age at first treatment, the relative risk in the period 5.0 to 24.9 years after first treatment remains significantly different from that for the period more than 25.0 years after first treatment ($\chi^2$ (1 df) = 8.94, $P<0.01$). In the period 5.0 to 24.9 years after treatment there is some tendency for the relative risk to be higher in patients treated at younger ages, but the trend with age is not quite significant ($\chi^2$ (1 df) = 3.38, $0.10>P>0.05$).

In Table VI the numbers of observed and expected deaths from neoplasms other than leukaemia and colon cancer in the periods 5.0 to 24.9 and more than 25.0 years after treatment are shown separately for males and females. For neither group is the relative risk in the later time period significantly increased. The relative risk in the period more than 25.0 years after first treatment remains significantly different from that for the earlier period after standardization for sex ($\chi^2$ (1 df) = 7.72, $P<0.01$).

To investigate further whether the apparent decrease in relative risk with time since exposure for neoplasms other

**Table V** Observed and expected deaths from neoplasms other than leukaemia or colon cancer at ages less than 85 years by time since first treatment and age at first treatment. Re-treated patients are included for 18 months after re-treatment.

| | *Time since first treatment (years)* | | | | | | | | | | | |
|---|---|---|---|---|---|---|---|---|---|---|---|---|
| | *5.0–24.9* | | | | *≧25.0* | | | | *Total ≧5.0* | | | |
| *Age at first treatment* | *O* | *E* | *O/E* | *Years at risk* | *O* | *E* | *O/E* | *Years at risk* | *O* | *E* | *O/E* | *Years at risk* |
| <25 | 13[a] | 6.59 | 1.97 | 16,519 | 13 | 12.32 | 1.06 | 5,597 | 26 | 18.90 | 1.38 | 22,117 |
| 25–34 | 73[c] | 47.19 | 1.55 | 40,294 | 61 | 62.50 | 0.98 | 12,181 | 134[a] | 109.69 | 1.22 | 52,475 |
| 35–44 | 135[c] | 98.37 | 1.37 | 30,432 | 75 | 68.06 | 1.10 | 6,919 | 210[c] | 166.42 | 1.26 | 37,351 |
| 45–54 | 114[b] | 84.15 | 1.35 | 12,870 | 28 | 22.40 | 1.25 | 1,602 | 142[c] | 106.56 | 1.33 | 14,472 |
| ≧55 | 50 | 43.09 | 1.16 | 4,131 | 1 | 1.28 | 0.78 | 71 | 51 | 44.38 | 1.15 | 4,201 |
| Total | 385[c] | 279.39 | 1.38 | 104,246 | 178 | 166.56 | 1.07 | 26,370 | 563[c] | 445.95 | 1.26 | 130,616 |

[a]$P<0.05$; [b]$P<0.01$; [c]$P<0.001$.

**Table VI** Observed and expected deaths from neoplasms other than leukaemia or colon cancer at ages less than 85 years in males and females by time since first treatment. Re-treated patients are included for 18 months after re-treatment.

| | *Time since first treatment (years)* | | | | | | | | | | | |
|---|---|---|---|---|---|---|---|---|---|---|---|---|
| | *5.0–24.9* | | | | *≧25.0* | | | | *Total ≧5.0* | | | |
| | *O* | *E* | *O/E* | *Years at risk* | *O* | *E* | *O/E* | *Years at risk* | *O* | *E* | *O/E* | *Years at risk* |
| Males | 331[c] | 236.97 | 1.40 | 86,713 | 154 | 145.69 | 1.06 | 21,870 | 485[c] | 382.66 | 1.27 | 108,583 |
| Females | 54[a] | 42.43 | 1.27 | 17,533 | 24 | 20.87 | 1.15 | 4,500 | 78[a] | 63.29 | 1.23 | 22,033 |
| Total | 385[c] | 279.40 | 1.38 | 104,246 | 178 | 166.56 | 1.07 | 26,370 | 563[c] | 445.95 | 1.26 | 130,616 |

[a]$P<0.05$; [c]$P<0.001$.

than leukaemia and colon cancer in the later part of the follow-up period could be due to confounding by age at exposure or sex, a Poisson regression modelling procedure (Darby *et al.*, 1985) was carried out in which the pattern of relative risk with time since treatment in 2.5 year intervals was examined after allowing simultaneously for the effect of age at first treatment and sex. No evidence of confounding was revealed.

*Cancer of the colon.* The relative risk for cancer of the colon was 1.30 (Table II, $P<0.05$). There was some tendency for the relative risk to decrease with increasing time since first treatment, but the trend was not quite significant (Table III; $\chi^2$ (1 df trend 5.0–7.4 years, . . . , ≧35.0 years) = 3.42, $0.10>P>0.05$). The relative risk was highest in the period 5.0–7.4 years after treatment with 5 deaths observed and 1.82 expected. During the period 5.0–24.9 years after treatment there were 28 deaths compared with 19.93 expected. This 40% increase was not quite significantly different from 0 ($0.10>P>0.05$) nor was it significantly different from the increase observed for all cancers other than leukaemia or colon cancer during this time period ($\chi^2$ (1 df) = 0.00, $P>0.10$).

*Leukaemia.* In healthy individuals receiving a single exposure to radiation, it seems unlikely, from consideration of the rate at which cells proliferate, that any death from radiation-induced cancer would occur within two years, and even in spondylitics in whom cell kinetics may be modified because they receive a series of fractional doses and are often anaemic before treatment, it seems very unlikely that it would occur within a year. In fact, after the three patients reported previously who died from leukaemia within four months of treatment and whose disease is thought to have been present at treatment (Smith & Doll, 1982), the next shortest latent interval occurred 15 months after a first course of treatment. It is impossible to be sure that this death was not due to irradiation and we therefore recalculated the person-years at risk including re-treated patients only for 12 months after re-treatment and counted observed deaths only for the same period. With this restriction there were 39 deaths from leukaemia compared with 12.29 expected from national mortality rates for England and Wales (Table II). This increase, which is statistically highly significant, ($P<0.001$), was greater, but not significantly so, in men than in women ($\chi^2$ (1 df) = 0.98, $P>0.10$). The relative risk was highest 2.5–4.9 years after treatment (Table III) and thereafter declined ($\chi^2$ (1 df trend 2.5–4.9 years, . . . ≧35.0 years) = 12.25; $P<0.001$). More than 15.0 years after treatment the relative risk was 1.87, and was still significantly increased ($P<0.05$). Its value during the period 15.0 to 24.9 years after treatment was similar to, but slightly less than, that for the period more than 25.0 years after treatment (1.80 against 1.94). Thus there is no evidence from these data that the increased risk disappeared completely, nor that it was changing materially more than 15.0 years after exposure.

There was no significant trend with age at exposure in the relative risk of leukaemia one or more years following exposure (Table VII. $\chi^2$ (1 df trend) = 0.66; $P>0.10$), nor was there any when the periods 1.0–14.9 years after exposure and more than 15.0 years after exposure were considered separately (1.0–14.9 years: $\chi^2$ (1 df trend) = 0.00, $P>0.10$; ≧15.0 years: $\chi^2$ (1 df trend) = 0.32, $P>0.10$). Within each age at first treatment group the relative risk more than 15.0 years after treatment was less than that in the period 1.0–14.9 years after treatment. Poisson regression modelling of the relative risk allowing simultaneously for time since treatment, age at treatment, and sex did not reveal any new features of the data.

Of the 36 deaths certified as due to leukaemia and occurring one or more years after first treatment, it was possible to obtain case notes for 32. For one of these, certified as due to acute myeloid leukaemia, the notes suggested a diagnosis of myelodysplasia. For 16 deaths, including 12 of the 14 certified as due to acute myeloid leukaemia, the case notes confirmed the type of leukaemia as

**Table VII** Observed and expected deaths from leukaemia at ages less than 85 years by age at first treatment. Re-treated patients are included for 12 months after re-treatment.

| | *Time since first treatment (years)* | | | | | | | | | | | |
|---|---|---|---|---|---|---|---|---|---|---|---|---|
| | *1.0–14.9* | | | | *≧15.0* | | | | *Total ≧1.0* | | | |
| *Age at first treatment (years)* | *O* | *E* | *O/E* | *Years at risk* | *O* | *E* | *O/E* | *Years at risk* | *O* | *E* | *O/E* | *Years at risk* |
| <25 | 1 | 0.34 | 2.98 | 14,479 | 0 | 0.58 | 0.00 | 13,161 | 1 | 0.91 | 1.10 | 27,640 |
| 25–34 | 5[b] | 1.00 | 4.98 | 34,446 | 5 | 2.26 | 2.21 | 30,503 | 10[b] | 3.26 | 3.06 | 64,949 |
| 35–44 | 8[c] | 1.20 | 6.66 | 27,224 | 4 | 2.77 | 1.44 | 20,061 | 12[c] | 3.97 | 3.02 | 47,285 |
| 45–54 | 4[a] | 1.03 | 3.90 | 13,020 | 5[a] | 1.50 | 3.34 | 6,396 | 9[b] | 2.52 | 3.57 | 19,416 |
| ≧55 | 4[a] | 0.83 | 4.85 | 5,325 | 0 | 0.39 | 0.00 | 1,148 | 4[a] | 1.22 | 3.28 | 6,472 |
| Total | 22[c] | 4.39 | 5.01 | 94,494 | 14[a] | 7.50 | 1.87 | 71,269 | 36[c] | 11.89 | 3.03 | 165,763 |

[a]$P<0.05$; [b]$P<0.01$; [c]$P<0.001$.

given on the death certificate. For a further 8 the type could be determined more precisely from the case notes than from the death certificate, but there was no conflict between the two. For 3 deaths the type of leukaemia was described less precisely in the case notes than on the death certificate, but there was no direct conflict. For the remaining 4 deaths (with certified types, acute lymphatic (1), chronic myeloid (1), and unspecified lymphatic (2)) the type of leukaemia described on the death certificate was contradicted by the case note information, and in 3 of these the case notes led to a diagnosis of acute myeloid leukaemia.

Review of the case notes and biopsy specimens of 5 of the 7 patients whose cause of death was given on the death certificate as aplastic anaemia showed that death was actually due to leukaemia in a further 2 patients (who died at 2 and 4 years after first treatment for spondylitis) (Court Brown & Doll, 1957; Smith & Doll, 1982). Leukaemia was also recorded on the death certificate, but not as the underlying cause of death, for 5 other patients (who died at 5, 6, 10, 12, and 32 years after first treatment) (Smith & Doll, 1982). No data for the study period as a whole are available for deaths in England and Wales with leukaemia mentioned on the death certificate but not as the underlying cause, nor is there information on the number of deaths from leukaemia that were certified as due to aplastic anaemia, or the number of deaths certified as due to leukaemia when leukaemia was not, in fact, the cause. We therefore assumed that the ratio of the number of such deaths to the number of deaths for which leukaemia was certified as the underlying cause was the same as we observed in the study series. To estimate the expected number of deaths with leukaemia mentioned on the death certificate we multiplied the expected number of deaths certified as due to leukaemia by 45/39 (see Table III). This procedure could not alter the overall estimate of relative risk from that given by the analysis based on leukaemia as an underlying cause only, but it could alter the estimate at different periods since treatment and, in fact, it increased the relative risk in the period between 1.0 and 14.9 years after treatment slightly from 5.01 to 5.53, with corresponding decreases earlier and later.

Observed and expected deaths one or more years after first treatment by type of leukaemia as recorded on the death certificate are shown in Table VIII. These results are difficult to interpret as the type was incompletely specified for one third of the observed deaths and for one fifth of those expected. Nearly half the deaths were certified as due to acute myeloid leukaemia. This is the specified type with the highest relative risk and the only one for which a significant increase was recorded overall ($P<0.001$), although there was a significant increase in deaths from chronic myeloid leukaemia in the period 1.0–14.9 years after treatment ($P<0.05$). Only two deaths were certified as due to chronic lymphatic leukaemia, and this was less than the number expected (2.38). For all types other than chronic lymphatic leukaemia, unspecified lymphatic leukaemia and unspecified chronic leukaemia, the relative risk in the period 1.0–14.9 years after first treatment course was greater than later, although for acute myeloid leukaemia the increase continued

**Table VIII** Observed and expected deaths from leukaemia at ages less than 85 years by type of leukaemia as recorded on the death certificate. Re-treated patients are included for 12 months following re-treatment.

| | *Time since first treatment (years)* | | | | | | | | |
|---|---|---|---|---|---|---|---|---|---|
| | *1.0–14.9* | | | *≧15.0* | | | *Total ≧1.0* | | |
| *Type of*[d] *leukaemia* | *O* | *E* | *O/E* | *O* | *E* | *O/E* | *O* | *E* | *O/E* |
| Acute myeloid | 7[c] | 1.42 | 4.93 | 10[c] | 2.92 | 3.42 | 17[c] | 4.34 | 3.91 |
| Acute lymphatic | 1 | 0.46 | 2.18 | 1 | 0.47 | 2.14 | 2 | 0.93 | 2.16 |
| Chronic myeloid | 3[a] | 0.65 | 4.61 | 0 | 1.40 | 0.00 | 3 | 2.05 | 1.46 |
| Chronic lymphatic | 0 | 0.54 | 0.00 | 2 | 1.84 | 1.09 | 2 | 2.38 | 0.84 |
| Unspecified acute | 2 | 0.39 | 5.07 | 0 | 0.39 | 0.00 | 2 | 0.79 | 2.54 |
| Unspecified chronic | 0 | 0.01 | 0.00 | 0 | 0.03 | 0.00 | 0 | 0.04 | 0.00 |
| Unspecified myeloid | 4[b] | 0.51 | 7.88 | 0 | 0.20 | 0.00 | 4[b] | 0.71 | 5.65 |
| Unspecified lymphatic | 2[a] | 0.28 | 7.08 | 1 | 0.09 | 10.77 | 3[b] | 0.38 | 8.00 |
| Unspecified leukaemia | 3[c] | 0.12 | 24.62 | 0 | 0.16 | 0.00 | 3[b] | 0.28 | 10.79 |
| All types | 22[c] | 4.39 | 5.01 | 14[a] | 7.50 | 1.87 | 36[b] | 11.89 | 3.03 |

[a]$P<0.05$; [b]$P<0.01$; [c]$P<0.001$; [d]ICD-8 codes are as follows: acute myeloid (205.0, 206.0, 207.2); acute lymphatic (204.0); chronic myeloid (205.1, 206.1); chronic lymphatic (204.1); unspecified acute (207.0); unspecified chronic (207.1); unspecified myeloid (205.9, 206.9); unspecified lymphatic (204.9); unspecified leukaemia (207.9).

to be statistically significant after 15 years (10 observed, 2.92 expected; $P<0.001$). The two deaths from chronic lymphatic leukaemia occurred 26 and 31 years after treatment, but even in the period more than 15 years after treatment they are only fractionally more than the number expected (1.84). Observed and expected deaths more than one year after first treatment by certified type of leukaemia and age at first treatment are shown in Table IX. For deaths certified as due to acute myeloid leukaemia the relative risk increased with increasing age at exposure. The number of deaths was small, however, and the trend was not quite statistically significant ($\chi^2$ (1 df) = 2.73; $0.10>P>0.05$). There is also a suggestion of a trend in relative risk with age at exposure for the 4 deaths certified as due to unspecified myeloid leukaemia, 2 of which were confirmed as acute myeloid leukaemia from the case notes.

*Diseases other than neoplasms*

There was a 51% increase in deaths from diseases other than neoplasms (Table II; $P<0.001$) and the relative risk for females was higher than that for males ($\chi^2$ (1df) = 7.09, $P<0.01$). The relative risk generally declined with increasing time since irradiation from 1.88 in the first five years to 1.20 more than 30 years after treatment (Table III; $\chi^2$ (1 df trend <2.5 years, ...≧35.0 years) = 40.70, $P<0.001$). More than 25.0 years after treatment there were 657 deaths compared with 493.85 expected. This 33% increase was statistically highly significant ($P<0.001$). Relative risks by age at first treatment were: <25 years, 2.09; 25–34 years, 1.56; 35–44 years, 1.51; 45–54 years, 1.51; and ≧55 years, 1.32. This decline in relative risk with age at first treatment was statistically highly significant ($\chi^2$ (1 df trend) = 14.14, $P<0.001$).

Following previous reports (Court Brown & Doll, 1965; Smith & Doll, 1982), the causes of death have been grouped into 4 classes (Table X). Class A consists of ankylosing spondylitis and other musculo-skeletal disorders, some of which might give rise to diagnostic confusion with spondylitis. A wider grouping of these diseases had to be considered than previously, due to changes from the 7th to the 9th revision of the ICD, which was used for the national rates in the last few years of follow-up. Class B consists of diseases that are clinically associated with ankylosing spondylitis (Hickling & Wright, 1983). No death was certified as due to amyloid disease, which is known to be a complication of spondylitis, although it was mentioned on 10 death certificates, and 57 deaths were attributed to nephritis (which is commonly due to unrecognized amyloid disease in spondylitics) against 18.42 expected. As in the earlier follow-up of this group, the numbers of deaths from every cause in Classes A and B (apart from amyloid disease) were significantly greater than expected ($P<0.001$ for each group except non-rheumatic chronic endocarditis, for which $P<0.01$). As expected, the largest proportionate increases were for ankylosing spondylitis, other musculo-skeletal disorders, and ulcerative colitis. For all diseases in Class A combined the ratio of observed to expected deaths was similar in men and women, but when ankylosing spondylitis was considered on its own, the ratio for women was significantly greater than that for men ($\chi^2$ (1 df) = 6.95, $P<0.01$). For each of the diseases in Class B the ratios took similar values in the two sexes, and the differences were not statistically significant ($P>0.10$ in each group).

Class C includes neoplasms and aplastic anaemia. The former have been commented on previously. The latter was the certified cause of 7 deaths, whereas less than one was expected (0.96). No further information beyond that given on the death certificates could be obtained for two patients who died respectively 18 and 20 years after exposure. Review of the notes and marrow biopsy specimens showed that two cases would have been better described as aleukaemic leukaemia (cases 32 and 41) (Court Brown & Doll, 1957) and that one (case 44) (Court Brown & Doll, 1957), which presented about two weeks after exposure, could be regarded as aplastic anaemia induced by treatment. Marrow specimens could not be obtained for either of the two remaining cases, but the information in the hospital notes suggested that alternative diagnoses would have been preferred (i.e. thrombocytopenia developing in a woman aged 48, 26 years after exposure and causing death 7 years later and macrocytic anaemia or possibly myelodysplasia presenting in a man aged 70, 37 years after exposure, causing death within a week). On this evidence, there does not seem to be any justification for believing that aplastic anaemia is liable to be a long term effect of irradiation, although conditions liable to be confused with it may well be.

Class D consists of all other causes of death, for which it was originally thought that mortality might be close to normal among patients with spondylitis (Court Brown & Doll, 1965) apart from a few extra deaths due to spinal injury. Previous studies (Court Brown & Doll, 1965; Smith & Doll, 1982), however, have reported an increase in all disease groups in this class, and this is confirmed by the present data based on longer follow-up (Table X). The proportionate increases for each of the individual diseases considered were less than for any of the diseases in Class B, other than amyloid disease. For all diseases in Class D combined, the ratio of observed to expected deaths was greater for women than for men ($\chi^2$ (1 df) = 7.00, $P<0.01$), but only for other circulatory disease was the difference statistically significant ($\chi^2$ (1 df) = 4.76, $P<0.05$).

**Table IX** Observed and expected deaths from leukaemia occurring more than one year after first treatment at ages less than 85 years by age at first treatment and type of leukaemia as recorded on the death certificate. Re-treated patients are included for 12 months following treatment.

| Type of leukaemia | <25 O | <25 E | <25 O/E | 25–34 O | 25–34 E | 25–34 O/E | 35–44 O | 35–44 E | 35–44 O/E | 45–54 O | 45–54 E | 45–54 O/E | ≧55 O | ≧55 E | ≧55 O/E | All ages O | All ages E | All ages O/E |
|---|---|---|---|---|---|---|---|---|---|---|---|---|---|---|---|---|---|---|
| Acute myeloid | 0 | 0.41 | 0.00 | 4 | 1.37 | 2.91 | 7 | 1.47 | 4.76 | 4 | 0.80 | 5.00 | 2 | 0.29 | 6.88 | 17 | 4.34 | 3.91 |
| Acute lymphatic | 0 | 0.11 | 0.00 | 1 | 0.28 | 3.62 | 0 | 0.28 | 0.00 | 1 | 0.18 | 5.63 | 0 | 0.09 | 0.00 | 2 | 0.93 | 2.16 |
| Chronic myeloid | 0 | 0.17 | 0.00 | 0 | 0.60 | 0.00 | 2 | 0.68 | 2.94 | 1 | 0.42 | 2.40 | 0 | 0.19 | 0.00 | 3 | 2.05 | 1.46 |
| Chronic lymphatic | 0 | 0.07 | 0.00 | 2 | 0.49 | 4.06 | 0 | 0.86 | 0.00 | 0 | 0.63 | 0.00 | 0 | 0.33 | 0.00 | 2 | 2.38 | 0.84 |
| Unspec. acute | 0 | 0.08 | 0.00 | 0 | 0.23 | 0.00 | 1 | 0.25 | 4.01 | 1 | 0.15 | 6.62 | 0 | 0.07 | 0.00 | 2 | 0.79 | 2.54 |
| Unspec. chronic | 0 | 0.00 | 0.00 | 0 | 0.01 | 0.00 | 0 | 0.01 | 0.00 | 0 | 0.01 | 0.00 | 0 | 0.01 | 0.00 | 0 | 0.04 | 0.00 |
| Unspec. myeloid | 0 | 0.04 | 0.00 | 0 | 0.16 | 0.00 | 1 | 0.23 | 4.35 | 1 | 0.16 | 6.08 | 2 | 0.11 | 17.81 | 4 | 0.71 | 5.65 |
| Unspec. lymphatic | 1 | 0.02 | 52.08 | 1 | 0.06 | 16.95 | 0 | 0.10 | 0.00 | 1 | 0.10 | 9.72 | 0 | 0.10 | 0.00 | 3 | 0.38 | 8.00 |
| Unspec. leukaemia | 0 | 0.02 | 0.00 | 2 | 0.06 | 31.90 | 1 | 0.09 | 10.88 | 0 | 0.07 | 0.00 | 0 | 0.04 | 0.00 | 3 | 0.28 | 10.79 |
| All types | 1 | 0.91 | 1.10 | 10 | 3.26 | 3.06 | 12 | 3.97 | 3.02 | 9 | 2.52 | 3.57 | 4 | 1.22 | 3.28 | 36 | 11.89 | 3.03 |

**Table X:** Observed and expected deaths at ages less than 85 years for males and females from causes other than neoplasms.

| Cause of death[d] | Males | | | Females | | | Total | | |
|---|---|---|---|---|---|---|---|---|---|
| | Observed | Expected | O/E | Observed | Expected | O/E | Observed | Expected | O/E |
| *Class A* | | | | | | | | | |
| Ankylosing spondylitis | 81[c] | 0.24 | 340.81 | 9[c] | 0.01 | 950.34 | 90[c] | 0.25 | 364.16 |
| Other musculo-skeletal disorders | 44[c] | 5.82 | 7.56 | 14[c] | 1.22 | 11.48 | 58[c] | 7.04 | 8.24 |
| Total | 125[c] | 6.06 | 20.62 | 23[c] | 1.23 | 18.68 | 148[c] | 7.29 | 20.29 |
| *Class B* | | | | | | | | | |
| Ulcerative colitis | 16[c] | 1.33 | 12.07 | 6[c] | 0.39 | 15.28 | 22[c] | 1.72 | 12.80 |
| Non-rheumatic chronic endocarditis | 18[b] | 8.63 | 2.09 | 2 | 1.47 | 1.36 | 20[b] | 10.10 | 1.98 |
| Amyloid disease | 0 | 0.40 | 0.00 | 0 | 0.06 | 0.00 | 0 | 0.45 | 0.00 |
| Nephritis | 48[c] | 15.69 | 3.06 | 9[b] | 2.73 | 3.30 | 57[c] | 18.42 | 3.09 |
| Pulmonary tuberculosis | 104[c] | 30.97 | 3.36 | 8[a] | 3.35 | 2.39 | 112[c] | 34.31 | 3.26 |
| Pneumonia | 137[c] | 75.06 | 1.83 | 35[c] | 16.45 | 2.13 | 172[c] | 91.51 | 1.88 |
| Other respiratory disease (incl. apical fibrosis) | 55[c] | 30.15 | 1.82 | 9[a] | 3.53 | 2.55 | 64[c] | 33.68 | 1.90 |
| Total | 378[c] | 162.23 | 2.33 | 69[c] | 27.98 | 2.47 | 447[c] | 190.19 | 2.35 |
| *Class C* | | | | | | | | | |
| All neoplasms | 623[c] | 464.86 | 1.34 | 104[a] | 82.35 | 1.26 | 727[c] | 547.21 | 1.33 |
| Aplastic anaemia | 6[c] | 0.77 | 7.84 | 1 | 0.19 | 5.16 | 7[c] | 0.96 | 7.30 |
| *Class D* | | | | | | | | | |
| Cerebrovascular disease | 176 | 157.09 | 1.12 | 55 | 44.79 | 1.23 | 231[a] | 201.88 | 1.14 |
| Other circulatory disease | 843[c] | 695.10 | 1.21 | 147[c] | 99.35 | 1.48 | 990[c] | 794.45 | 1.25 |
| Bronchitis | 169[c] | 123.32 | 1.37 | 9 | 9.23 | 0.97 | 178[c] | 132.55 | 1.34 |
| Peptic ulcer | 26 | 20.66 | 1.26 | 6[a] | 1.90 | 3.15 | 32[a] | 22.56 | 1.42 |
| Other gastro-intestinal disease | 49[c] | 26.23 | 1.87 | 12[a] | 6.52 | 1.84 | 61[c] | 32.75 | 1.86 |
| Violence | 108[a] | 86.18 | 1.25 | 19[a] | 10.46 | 1.82 | 127[b] | 96.65 | 1.31 |
| All other causes | 132[c] | 85.78 | 1.54 | 41[c] | 21.13 | 1.94 | 173[c] | 106.92 | 1.62 |
| Total | 1,503[c] | 1,194.36 | 1.26 | 289[c] | 193.38 | 1.49 | 1,792[c] | 1,387.76 | 1.29 |
| Cause unknown | 2 | | | 1 | | | 3 | | |

[a]$P<0.05$; [b]$P<0.01$; [c]$P<0.001$; [d]ICD-7 codes are as follows: Class A: ankylosing spondylitis (722.1); other musculoskeletal disorders (rest of 720–749). Class B: ulcerative colitis (572.2); chronic endocarditis (421); amyloid disease (289.1); nephritis (590–594); pulmonary tuberculosis (001–008); pneumonia (490–493); other respiratory disease (rest of 470–527). Class C: all neoplasms (140–239); aplastic anaemia (292.4). Class D: cerebrovascular disease (330–334); other circulatory disease (rest of 400–468); bronchitis (500–502, 526); peptic ulcer (540–542); other gastrointestinal disease (rest of 530–587); violence (800–999).

**Table XI:** Observed and expected deaths from all causes except neoplasms at age less than 85 years for males and females by age at observation and calendar period. Re-treated patients are included for 18 months after re-treatment.

| Calendar period | Age at observation | | | | | | | | | | | | |
|---|---|---|---|---|---|---|---|---|---|---|---|---|---|
| | <25 | 25– | 30– | 35– | 40– | 45– | 50– | 55– | 60– | 65– | 70– | 75– | 80–84 |
| Up to 1 Jan. 1978 | | | | | | | | | | | | | |
| Observed | 13 | 47 | 57 | 75 | 123 | 176 | 238 | 305 | 309 | 247 | 172 | 133 | 74 |
| Expected | 8.69 | 15.50 | 22.60 | 34.52 | 55.47 | 91.32 | 137.08 | 176.33 | 203.21 | 193.13 | 157.44 | 104.21 | 56.81 |
| O/E | 1.50 | 3.03 | 2.52 | 2.17 | 2.22 | 1.93 | 1.74 | 1.73 | 1.52 | 1.28 | 1.09 | 1.28 | 1.30 |
| 1 Jan. 1978 to 1 Jan. 1983 | | | | | | | | | | | | | |
| Observed | 0 | 0 | 0 | 0 | 1 | 2 | 19 | 38 | 70 | 85 | 101 | 73 | 36 |
| Expected | 0 | 0 | 0 | 0 | 0.31 | 3.04 | 13.68 | 33.96 | 50.68 | 74.28 | 73.84 | 56.11 | 23.98 |
| O/E | — | — | — | — | 3.23 | 0.66 | 1.39 | 1.12 | 1.38 | 1.14 | 1.37 | 1.30 | 1.50 |
| Total | | | | | | | | | | | | | |
| Observed | 13 | 47 | 57 | 75 | 124 | 178 | 257 | 343 | 379 | 332 | 273 | 206 | 110 |
| Expected | 8.69 | 15.50 | 22.60 | 34.53 | 55.78 | 94.36 | 150.76 | 210.29 | 253.89 | 267.41 | 231.28 | 160.32 | 80.79 |
| O/E | 1.50 | 3.03 | 2.52 | 2.17 | 2.22 | 1.89 | 1.70 | 1.63 | 1.49 | 1.24 | 1.18 | 1.28 | 1.36 |

### Discussion

### *Neoplasms other than leukaemia or colon cancer*

Previous analyses of the mortality among patients with ankylosing spondylitis treated with X-rays (Court Brown & Doll, 1957; Smith & Doll, 1982) have shown, in conjunction with many other studies, that exposure to substantial doses of ionizing radiation can cause cancer in nearly every organ in the body. From the earlier follow-up of the spondylitic population the relative risk for cancers of heavily irradiated sites was found to have declined more than 20 years after treatment but the decline was not statistically significant. The extended follow-up of this cohort has indicated that, for that group of cancers, and also for the larger group of all neoplasms other than leukaemia or colon cancer, the period of increased risk between 5.0 and 24.9 years after irradiation is followed by a period in which the observed number of deaths is close to that expected from national rates. For a smaller group of patients with ankylosing spondylitis, who were diagnosed during the same period as patients in this series, but who were not treated with X-rays, the number of observed deaths from cancer was almost exactly equal to that expected using national rates (Smith *et al.*, 1977). Thus the present results suggest that radiation treatment increases the risk of death from cancer during a period that starts about 5 years after exposure, and that the proportionate increase in risk reaches a maximum about 10 years after the exposure and then gradually decreases.

This is the first large study to suggest an apparent end to the effects of exposure to radiation for neoplasms other than leukaemia and the possibility must be considered that the findings are spurious. We have already shown that this result cannot be explained as due to the variation with time in the types of cancer observed nor to the progressive attenuation of the older members of the population irradiated relatively late in life. There is also evidence that patients who survived and remained in the study for 25 years after the initial treatment received doses that were no lower than those who did not. The average estimated total body dose in patients who remained in the study for less than 25.0 years is 1.87 Gy (187 rad), while for those who remained in the study for longer it is 2.04 Gy (204 rad). Inadequate ascertainment of death in the later period of follow-up, resulting in the erroneous assumption that some patients who had died were still alive, would also cause the appearance of a spurious decrease in relative risk with increasing time since treatment. In order to investigate the possibility that this might have happened, mortality from all causes of death other than neoplasms was tabulated by age at observation and calendar-period (see Table XI). For the total follow-up period, the relative risk was greatest in individuals in their late twenties in whom it reached a value of 3.03 and then declined. However the decline did not continue into old age, and above the age of 65 the relative risk remained approximately constant with age, at about 1.24. In the last five calendar years of follow-up the pattern was similar to that seen in earlier years, and in the 65–84 age group the relative risk was 1.29, slightly greater than the value of 1.22 seen in previous years (Table XI). These findings provide no evidence of inadequate ascertainment of death in the oldest age groups in this study.

After the initial course of treatment it seems likely that the relative risk from neoplasms other than leukaemia or colon cancer was inflated for about 5 years by tumours that caused symptoms for which the treatment was given. A similar pattern might be expected after re-treatment, and following re-treated patients for a further 18 months only may have caused a slight underestimation in relative risk. For re-treated patients, however, the average time between the first and second treatments was only 2.0 years and only 6 patients were known to have received a second treatment more than 22 years after the first. The fact that we have not followed re-treated patients for a full 5 years after their second treatment cannot therefore have affected the results in the period more than 22 years after the initial treatment. Thus, investigation has not revealed any evidence to suggest that the present findings can be explained in any way other than by the effect of the irradiation having effectively ceased or, at least, materially diminished.

It does not necessarily follow that the same pattern of relative risk with time since exposure will hold for every type of cancer other than leukaemia, and there is already some evidence of heterogeneity between the patterns for the individual sites. A parallel analysis has been carried out of data from the earlier follow-up of this group of patients with mortality up to the end of 1978 in Japanese atomic bomb survivors included in the Life Span Study (LSS) who received total doses of at least 1 Gy (100 rad) (Darby *et al.*, 1985). In that analysis, when mortality from a selected group of solid tumours was compared with mortality from those same sites in the LSS, the trends in relative risk with time since exposure were not significantly different, and when data from the two studies were combined, they were consistent with a model in which the relative risk did not vary between about 5 and at least 30 years following exposure. The sites selected for this comparison consisted of pharynx, oesophagus, stomach, pancreas, larynx, lung, ovaries, skin, and bones (excluding jaw and nose). In the LSS, however, for neoplasms other than leukaemia or tumours of the selected sites, the relative risk of mortality increased with time since exposure, even after adjusting for age at exposure (Darby *et al.*, 1985), and the overall trend was significantly different from that seen in the two studies for the selected sites. Although the numbers of deaths were too few for the increasing trend in any individual site of cancer to be significant statistically, cancers of the bladder, colon, and liver and gall-bladder were the chief contributors (Darby *et al.*, 1985). Three of the four deaths from multiple myeloma in the high dose group also occurred more than 25 years after exposure (Radiation Effects Research Foundation, 1980). In the spondylitis patients multiple myeloma and cancers of the bladder and liver were among the few types of cancer for which higher relative risks were observed more than 25 years after exposure than in the earlier period (see Table IV).

In a large international study of the incidence of second cancers in women treated with radiation for cervical cancer, the relative risk for cancers of sites close to and at intermediate distances from the cervix also increased significantly with time since treatment, and the increasing trend lasted for at least 30 years following exposure (Boice *et al.*, 1985). There were significant increases in relative risk with time since exposure for a number of individual cancer types including multiple myeloma and bladder cancer. Thus all three studies are consistent with a very long term increase in risk following exposure to radiation for these two diseases. The remaining sites of cancer for which the relative risk increased significantly with time since exposure in the cervix cancer patients were *corpus uteri*, ovary, and rectum. For the first two of these sites there is little information in the spondylitis series as it includes so few women, while for the rectum there was little evidence of an increase in any time period. Thus some, at least, of the apparent discrepancies between the overall patterns of relative risk following radiation exposure found in three large studies may have arisen because different groups of cancer types were examined in each study, and they disappear when the individual types of cancer are considered. When all three studies are considered together there are now starting to be enough data to distinguish different temporal patterns of radiogenic risk between the different cancer types as well as for leukaemia.

Age at irradiation did not, in our study, significantly affect the relative risk of mortality fron neoplasms other than leukaemia or colon cancer, although during the period 5.0 to 24.9 years since exposure the relative risk did fall progressively from 1.97 at ages under 25 years to 1.16 at age over 55 years (Table V). In the LSS a significant decrease in relative risk with increasing age at exposure occurred, but this was chiefly due to the high relative risk in those aged under 15 at exposure, with only a slight trend apparant among those exposed at older ages (Darby *et al.*, 1985). In the spondylitic series there were no patients aged under 10 at exposure, and only 16 in the age range 10–14. At older ages the two sets of data are in close agreement.

*Leukaemia*

The extended follow-up of the spondylitis patients has confirmed the earlier finding that the increase in risk of mortality from leukaemia reaches its maximum within 5 years of commencement of radiotherapy, and then declines (Smith & Doll, 1982). This finding is consistent with results of the LSS, although in that study follow-up did not begin until October 1950, so no data for this cohort are available for the first 5 years after the bombings. The earlier spondylitis data (Smith & Doll, 1982) were consistent with the increase in leukaemia having ceased by 18 years after treatment, but the extended follow-up indicates that the period of increased risk is longer than this (Table VIII) and is in agreement with results from the LSS where the relative risk more than about 30 years after exposure is still around 2 (Darby *et al.*, 1985). The finding that the relative risk more than 25.0 years after the first treatment is slightly greater than in the previous 10 years is unexpected, and could be due to the play of chance, as the total numbers of deaths from leukaemia in the periods 15–24 years and more than 25 years after treatment are each only 7. Another possible explanation might be that some of the leukaemias occurring in the later period of follow-up were due to treatment of the spondylitis by drugs as at least one of those that are commonly used (phenylbutazone) is possibly leukaemogenic (International Agency for Research on Cancer, 1977). It should be noted too that the only death certified as due to leukaemia which on review of the clinical and haematological evidence appeared not to be due to the disease, occurred 32 years after first irradiation. The further follow-up has confirmed the earlier finding that neither the magnitude nor the temporal pattern of the leukaemia relative risk are greatly influenced by the age of the patient when first treated (Table VII).

It is impossible to draw firm conclusions from the analysis of observed and expected deaths by type of leukaemia as recorded on the death certificate, due to the large proportion of both observed and expected deaths for which the type was incompletely specified. The results are, however, in accordance with earlier suspicions that the relative risk of acute myeloid leukaemia following exposure to radiation may be greater than for other types of leukaemia (Darby, 1985) and they confirm the belief that chronic lymphatic leukaemia is much less readily inducible by radiation than the other types (Court Brown & Doll, 1965; Darby, 1985). It seems likely that the relative risk of acute myeloid leukaemia increases with age at exposure whereas there is no such tendency for this to occur with the other types (Table IX). When expressed in terms of excess risk there is also a steep rise in rate with increasing age at exposure for acute myeloid leukaemia and little or none for other leukaemias. (The excess risks per 100,000 years at risk in age at treatment groups: <25 years, 25–34 years, 35–44 years, 45–54 years, and ≧55 years were: −1.5, 4.0, 11.7, 16.5 and 26.4 for deaths certified as acute myeloid leukaemia, and 2.0, 6.6, 3.7, 12.6 and −12.7 for all other types of leukaemia apart from unspecified myeloid leukaemia.) These findings are in accord with recently published data from the LSS in which the annual incidence rate of acute myeloid leukaemia among those with total dose of at least 1 Gy (100 rad) increases steeply with age at exposure from 2.4 per 100,000 in those aged under 15 at exposure to 36.8 in those aged over 45, whereas there was a slight decrease in incidence with increasing age at exposure for other types of leukaemia (Darby, 1985; Ichimaru *et al.*, 1981). In the spondylitis patients 47% of leukaemia deaths were certified as due to acute myeloid leukaemia, and the preferred diagnosis was acute myeloid leukaemia in two thirds of those whose case notes were reviewed. In the Japanese series, in contrast, among those with total doses of at least 1 Gy (100 rad), a definite diagnosis of acute myeloid leukaemia was made in only 36% of leukaemia deaths (with a further 20% in which the diagnosis is described as other types of acute leukaemia). The higher proportion of leukaemia deaths due to the acute myeloid type in the spondylitis series compared with the LSS accounts for the steeper rise in excess leukaemia risk with age at exposure in the spondylitics than in the atomic bomb survivors that has been reported in previous comparisons of the two studies (Darby *et al.*, 1985; Ichimaru *et al.*, 1978; Smith & Doll, 1982).

*Diseases other than neoplasms*

For diseases other than neoplasms, the pattern of relative risk following treatment is different from that for neoplastic disease, with an increased level of risk still apparent 30 years after first treatment (Table III). The high mortality is not confined to those diseases that have long been recognized clinically to be associated with spondylitis, but was observed also for all other groups of diseases, though to a lesser extent. A similar excess has, however, also been observed in unirradiated patients (Radford *et al.*, 1977) and should not be attributed to the treatment. This conclusion is strengthened by our finding that when the data were subdivided by age at observation the relative risk rose from 1.50 for subjects under 25 years of age to over 3 among those aged 25–29 and remained at above 2 until age 40–44 before declining (Table XI). Since the age at clinical onset of spondylitis is, in the vast majority of cases, between 15 and 35 (Kelsey, 1982) the ages at which the relative risk was highest lag the ages at which onset is most likely to occur by about 10 years. In contrast, when the data were subdivided by time since first treatment, the relative risk tended to decline progressively from the time of first treatment and never exceeded 2 (Table III). The increased risk for non-neoplastic disease is, therefore, more closely associated with the patient's age than with the time since first radiotherapy treatment.

The relative risk for diseases other than neoplasms was higher in women than in men (Table II), but only 16.5% of this irradiated series were women, compared with 37% of the group of unirradiated spondylitics diagnosed during the same period (Smith *et al.*, 1977). These differences may reflect a reluctance to recommend radiation treatment for women of childbearing age even before 1955, so that even though the disease is generally milder in women than in men (Kelsey, 1982; Radford *et al.*, 1977), it is worse in the irradiated series because of the selection of more severe cases for treatment.

We thank the directors and staffs of the 87 British radiotherapy departments for their continued cooperation in this study, Dr C.A. Lewis with whom we are collaborating to estimate the radiation doses to individual organs, and Professor D.J. Weatherall for help in the review of case notes. The task of tracing the members of the study population was undertaken by staff of the National Health Service Central Registers, Mary Burgess, Kate Hughes, and Valerie Weare and we much appreciate all their assistance. Help with coding and computing has been given by Elizabeth Arms, Eva Darby, Freda Fearn, Jackie O'Hagan and Irene Stratton. The manuscript was typed by Cathy Harwood. The study was funded initially by the Medical Research Council and is now supported by the Imperial Cancer Research Fund. Funding for re-coding the original data to record exact dates, rather than years, was provided by the National Radiological Protection Board.

### References

BOICE, J.D., DAY, N.E., ANDERSON, A. & 33 others (1985). Second cancers following radiation treatment for cervical cancer. An international collaboration among cancer registries. *J. Natl Cancer Inst.*, **74**, 955.

CASE, R.A.M., COGHILL, C., DAVIS, J.M. & 5 others (1976). *Serial Mortality Tables: Neoplastic Disease, 1, England and Wales 1911–1970*. Division of Epidemiology, Institute of Cancer Research: London.

COURT BROWN, W.M. & DOLL, R. (1957). *Leukaemia and Aplastic Anaemia in Patients Irradiated for Ankylosing Spondylitis*. HMSO: London.

COURT BROWN, W.M. & DOLL, R. (1959). Adult leukaemia. Trends in mortality in relation to aetiology. *Br. Med. J.*, **1**, 1063.

COURT BROWN, W.M. & DOLL, R. (1965). Mortality from cancer and other causes after radiotherapy for ankylosing spondylitis. *Br. Med. J.*, **2**, 1327.

COX, D.R. & HINKLEY, D.V. (1974). *Theoretical Statistics*. Chapman and Hall: London.

DARBY, S.C. (1985). The value of subtyping in studies of irradiation and human leukaemia. *Leukaemia Res.*, **9**, 699.

DARBY, S.C., NAKASHIMA, E. & KATO, H. (1985). A parallel analysis of cancer mortality among atomic bomb survivors and patients with ankylosing spondylitis given x-ray therapy. *J. Natl Cancer Inst.*, **72**, 1.

HICKLING, P. & WRIGHT, G. (1983). Seronegative arthritis. In *Oxford Textbook of Medicine*, Weatherall, D.J. *et al.* (eds). Oxford University Press: Oxford.

ICHIMARU, M., ISHIMARU, T., MIKAMI, M. & 2 others (1981). Incidence of leukaemia in a fixed cohort of atomic bomb survivors and controls, Hiroshima and Nagasaki, October 1950–December 1978. *Technical Report RERF TR13–81*. Radiation Effects Research Foundation: Hiroshima.

INTERNATIONAL AGENCY FOR RESEARCH ON CANCER (1977). *Monograph 13. Some Miscellaneous Pharmaceutical Substances*. International Agency for Research on Cancer: Lyon.

KELSEY, J.L. (1982). *Epidemiology of Musculo-skeletal Disorders*. Oxford University Press: New York.

MACMAHON B., COLE, P. & BROWN, J. (1973). Etiology of human breast cancer. *J. Natl Cancer Inst.*, **50**, 21.

OFFICE OF POPULATION CENSUSES AND SURVEYS (1975). *Cancer Mortality. England and Wales 1911–1970*. HMSO: London.

OFFICE OF POPULATION CENSUSES AND SURVEYS (1979). *Life Tables*. Series DS no. 2. HMSO: London.

PEARSON, E.S. & HARTLEY, H.O. (1976). *Biometrika Tables for Statisticians, Vol. I.* Biometrika Trust: London.

RADFORD, E.P., DOLL, R. & SMITH, P.G. (1977). Mortality among patients with ankylosing spondylitis not given X-ray therapy. *New Engl. J. Med.*, **297**, 572.

RADIATION EFFECTS RESEARCH FOUNDATION (1980). *Life Span Study Report 9, 1950–1978. Supplementary Tables*. Radiation Effects Research Foundation: Hiroshima.

SMITH, P.G. & DOLL, R. (1978). Age- and time-dependent changes in the rates of radiation-induced cancers in patients with ankylosing spondylitis following a single course of X-ray treatment. In *Late Effects of Ionizing Radiation. Vol. I.* p. 205. International Atomic Energy Agency: Vienna.

SMITH, P.G. & DOLL, R. (1982). Mortality among patients with ankylosing spondylitis after a single treatment course with X-rays. *Br. Med. J.*, **1**, 449.

SMITH, P.G., DOLL, R. & RADFORD, E.P. (1977). Cancer mortality among patients with ankylosing spondylitis not given X-ray therapy. *Br. J. Radiol.*, **50**, 728.

TOKUNAGA, M., LAND, C.E., YAMAMOTO, T. & 4 others (1984). Incidence of female breast cancer among atomic bomb survivors, Hiroshima and Nagasaki, 1950–1980. *Technical Report RERF TR15–84*. Radiation Effects Research Foundation: Hiroshima.

WORLD HEALTH ORGANIZATION (1957). *Manual of the International Statistical Classification of Diseases, Injuries and Causes of Death*. 7th revision. WHO: Geneva.

WORLD HEALTH ORGANIZATION (1967). *Manual of the International Statistical Classification of Diseases, Injuries, and Causes of Death*. 8th revision. WHO: Geneva.